# Consumer Sensory Perceptions of Natural Ingredients: A Multi-Country Comparison

**DOI:** 10.3390/foods14101775

**Published:** 2025-05-16

**Authors:** Jisoo Choi, Edgar Chambers, Jeehyun Lee

**Affiliations:** 1 Department of Food Science and Nutrition, College of Human Ecology, Pusan National University, Busan 46241, Republic of Korea; 2 Center for Sensory Analysis and Consumer Behavior, Kansas State University, Manhattan, KS 66502, USA

**Keywords:** natural, ingredient, consumer, perception, cross-cultural, check-all-that-apply

## Abstract

Consumer perceptions of the term ‘natural’ do not always align with those of science or public policy. Further examination of the term from a consumer standpoint is therefore necessary. In this study, we aimed to identify differences in consumer perceptions of natural ingredients across various countries and demographic segments to determine whether a common concept of natural ingredients exists. Twenty ingredients were assessed to identify those perceived as natural by consumers. A total of 8191 consumers (about 630 consumers per country) were surveyed. Cluster analysis identified four clusters of countries with similar perceptions of natural ingredients. Most ingredients were evaluated similarly across countries; however, specific ingredients differed among certain countries. Several ingredients considered natural according to scientific, public policy, or marketing standards were not perceived as natural by consumers. These insights into perceptions of natural ingredients in each country may help governments, public policy experts, and food manufacturers reconsider strategies for marketing natural products and educating consumers about natural ingredients.

## 1. Introduction

The natural and organic products industry has expanded tremendously, growing from USD 97 billion in 2007 to over USD 300 billion in 2023, demonstrating consistent annual growth. Consumer sales exceeded USD 250 billion in 2020, with an average annual growth rate of 8% over the previous decade [1]. This upward trend has reflected the transition of natural products from niche markets to widespread mainstream adoption [1,2]. However, whether consumers fully comprehend the meaning of the term “natural” on food labels remains uncertain.

Within the food industry, the term “natural” has been used extensively despite the absence of a clear definition, and many products are not subject to regulation by a competent authority [3]. Manufacturers frequently display the term “natural” on product packaging even when the items contain artificial ingredients. The 2015 Consumer Reports Natural Food Labels Survey [4] highlighted consumer confusion stemming from inconsistent labeling, which led several organizations to initiate legal action against food companies for using potentially misleading claims. These cases specifically targeted terms such as “natural” and “healthy”, alleging that their usage was deceptive.

Food safety experts from Consumer Reports supported these concerns, characterizing the use of the word “natural” as a marketing tactic designed to mislead consumers. They further emphasized that regulators should either prohibit the term entirely or establish a uniform definition [4]. Even in the absence of strategic marketing, inherent consumer bias has been repeatedly demonstrated in studies showing a preference for “natural” over terms such as “artificial” or “chemical” [3]. Accordingly, scientists, manufacturers, retailers, and regulatory agencies must continue to collaborate to enhance consumer understanding of potential risks related to food choices. Given the commercial importance of the term “natural” to brands, manufacturers, and other stakeholders, it is essential to monitor shifts in consumer behavior and gain a comprehensive understanding of how preferences evolve over time [5].

In December 2015, the Consumer Reports National Research Center conducted a survey involving 1005 adults to assess their views on natural food labeling. Sixty-two percent of respondents reported that they typically purchased food labeled as natural. Most believed that the term should imply the absence of chemical processing (85%), artificial ingredients or colors (84%), toxic pesticides (84%), and genetically modified organisms (82%). Despite consumer reliance on the term in purchasing decisions, regulatory oversight remains limited. The Consumers Union [6] petitioned the U.S. Food and Drug Administration (FDA) to restrict the use of the term “natural” on food labels in order to reduce consumer misunderstanding. In response, the FDA [7] collected public input, which revealed a wide range of interpretations, including perceptions that natural denotes “healthy” or that “not fresh should not be considered healthy”. The FDA [8] also invited comments on whether the term should be officially defined. Although no definition has been formally adopted, the FDA has traditionally interpreted “natural” to mean that “nothing artificial or synthetic has been included in, or has been added to, a food that would not normally be expected to be in that food”.

Román [9] noted that the concept of naturalness in food is inherently abstract and often linked to health, freshness, and the perception that products are organically or locally sourced. Moreover, studies examining naturalness in foods have reported considerable variation in the composition of such foods and inconsistency in how the term is defined by different authors [9,10,11,12,13,14]. While research on consumer perceptions of “natural” has primarily focused on the United States, the term remains widely used in numerous countries without regulatory clarity [10]. Furthermore, the term can be misleading, as certain naturally occurring compounds, such as arsenic and *Clostridium botulinum* toxin, are highly harmful to human health. Similarly, excessive intake of natural nutrients, such as Vitamin A, may result in fatal outcomes.

However, comparative studies evaluating consumer interpretations of naturalness across different regions are limited. In particular, few studies have examined how demographic variables influence perceptions of what is considered natural. Most existing research has focused on Western, Educated, Industrialized, Rich, and Democratic (WEIRD) populations, resulting in a significant knowledge gap with respect to consumer behavior in non-WEIRD countries [11]. This gap also complicates efforts to accurately interpret cross-cultural consumer psychology and decision-making processes [12].

In this study, we aimed to identify whether specific ingredients are perceived as natural by consumers from 13 countries, including both WEIRD and non-WEIRD regions. Additionally, the study examined how perceptions of naturalness vary by demographic characteristics, including age, sex, education level, and the number of adults or children in the household.

## 2. Materials and Methods

### 2.1. Participant Profiles

Participants (n = 8191) were recruited via the online survey platform Qualtrics (Provo, UT, USA) from existing consumer databases in 13 countries (n = 630 per country, with 631 in India). Each database contained approximately 1,000,000 potential respondents. The selected countries were Australia, Brazil, China, India, Japan, Mexico, Peru, Russia, South Africa, Spain, Thailand, the United Kingdom (UK), and the United States of America (USA). These countries represented all permanently inhabited continents (North America, South America, Europe, Africa, Asia, and Oceania) and included both WEIRD and non-WEIRD populations with varied dietary habits. Eligibility was further determined by the size of each country’s database and the anticipated survey response rate. For instance, Egypt and Ghana were initially tested but excluded from the final sample due to low response rates. Ghana returned fewer than 300 responses, and Egypt yielded minimal participation in the 55+ age group. Data from the USA were not collected as part of this study but were sourced from a previously published study by one of the authors for comparative purposes. The demographic profile for each country was balanced by sex (50% male, 50% female) and by age (33% each for 18–34, 35–54, and 55+ years). Information on education level and household composition (number of adults and children) was also collected; although, this information was not balanced by sex, age, or country (Table 1). Participants were compensated by Qualtrics using its standard reward system.

### 2.2. Questionnaire

The questionnaire assessed consumer perceptions regarding whether specific ingredients (Table 2) were considered natural. A check-all-that-apply (CATA) format was used, whereby participants marked each ingredient they perceived as natural. The 20 tested ingredients were derived from a previously published USA questionnaire [13]. All ingredients were available or approved in the respective countries. All participants completed all questions with no branching logic, and the ingredient order was randomized for each respondent.

If English was not the respondent’s primary language, the questionnaire was translated into the respective language (Brazilian Portuguese, Mandarin Simplified Chinese, Hindi, Japanese, Spanish, Russian, Afrikaans, or Thai). A modified version of the translation, review, adjudication, pretesting, and documentation (TRAPD) method [14,15] was followed.

The initial translation was conducted by a native speaker with subject-matter expertise and proficiency in English. A second subject-matter expert conducted a back-translation and made necessary revisions. Discrepancies between the original and the back-translated version were addressed, whether due to translation errors or other inconsistencies [16,17].

The two translators for each language reviewed the final version collaboratively, either in person or online, to ensure semantic equivalence. This approach has been applied in other multilingual surveys [18,19,20,21]. A soft launch involving 50 participants per country was conducted to assess clarity and timing. The translated questionnaires [14] were verified for comprehensibility and completion feasibility.

Soft launch data were monitored, and no missing responses were detected. Responses to validity check items designed to evaluate attentiveness were appropriately scored. In India and South Africa, participants could choose between English and the translated version, given that a substantial portion of the population regularly uses English and may not use the other offered language (e.g., Afrikaans in South Africa).

### 2.3. Data Analysis

Frequencies and percentages were calculated using Excel (Microsoft Office LTSC Professional Plus 2021). XLSTAT ver. 2024.4.0 (Addinsoft, New York, NY, USA) was used to analyze CATA data for the 20 ingredients across 13 countries. Multiple comparisons were performed using the critical difference (Sheskin) procedure to identify significant differences in ingredient perception between countries (*p* < 0.05).

The Kruskal–Wallis test, a non-parametric method suitable for comparing two or more independent samples [22], was used to assess differences in mean naturalness perception across age groups (18–34, 35–54, 55+). The Mann–Whitney U test was applied to compare perception differences by education level (primary/less with high school vs. college/university), sex (male vs. female), number of adults in the household (1–2 vs. 3 or more), and presence of children under 18 years (none vs. one or more) [22].

SAS 9.4 (SAS Institute Inc., Cary, NC, USA) was used to perform cluster analysis, grouping countries based on perception frequency. Correspondence analysis was conducted to visualize the results.

## 3. Results

### 3.1. Overall Perceptions of 20 Ingredients as Natural

Overall, consumers differed in their perceptions of the various ingredients as natural (Table 2). Three ingredients, corn (73.10%), wheat flour (69.77%), and soybeans (69.05%), were most frequently thought to be natural by consumers. Only two other ingredients, pea flour (58.77%) and salt (57.22%), were considered natural by most consumers. Lecithin (8.69%), sodium acid pyrophosphate (SAPP; 2.04%), and butylated hydroxyanisole (BHA; 1.43%) were perceived as natural by less than 10% of consumers.

### 3.2. Consumer Perception Differences for 20 Ingredients According to Country

The participants’ perceptions of whether the 20 ingredients were natural are shown for each country in Table 3. More than 50% of the respondents in every country identified no single ingredient as natural. However, corn (excluding Thailand), wheat flour (excluding Brazil), and soybeans (excluding Russia and the UK) were considered as natural by 50% or more of the consumers in most countries. BHA and SAPP were not considered natural (less than 5%) by consumers in any country. All other ingredients showed a range of differences across countries. Black beans, canola oil, maltodextrins, and sorghum flour showed the largest differences with at least one country having over 50% of consumers agree that the ingredient was natural; whereas, in one other country, less than 10% of respondents agreed that the ingredient was natural. Evidently, the concept of natural is perceived differently across the 13 countries. Although the reasons for consumers’ determinations of naturalness were not explored in this study, the differences likely occurred because of differences in familiarity with the ingredient, perceived healthfulness, or the chemical-sounding name of the ingredient in various countries. Additionally, clear differences were evident in the perception of ingredients, such as baking soda and sodium bicarbonate, which are the same ingredient with common and chemical names. Those differences likely occurred because of familiarity, countries using different names for these ingredients, and the chemical name sounding less natural in some contexts. Differences were also observed in consumer perceptions of insect powder, canola oil, maltodextrins, and molasses. 

### 3.3. Consumer Perceptions of 20 Ingredients According to Country Clusters

**Figure 1:**
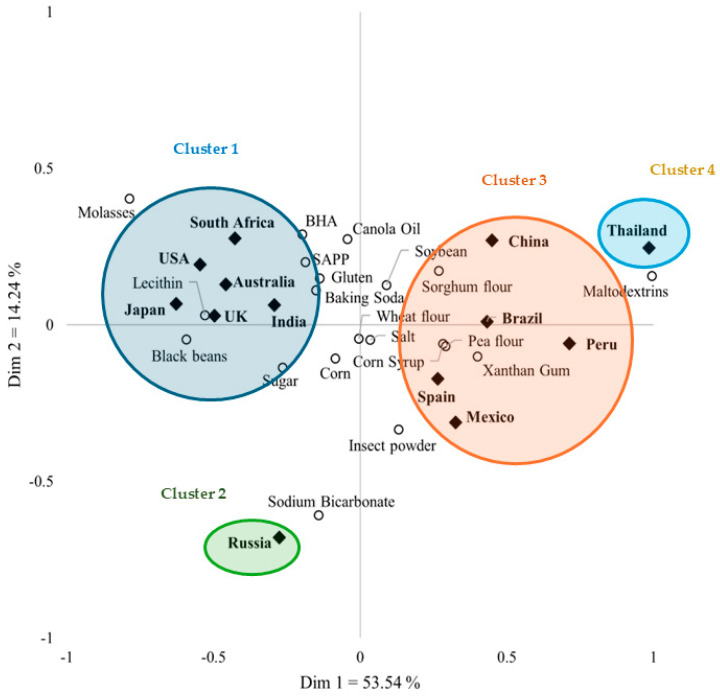
Correspondence analysis showing the similarities and differences between 20 ingredients considered as natural by consumers in 13 countries. Abbreviations: USA, United States of America; UK, United Kingdom; SAPP, sodium acid pyrophosphate; and BHA, butylated hydroxyanisole.

Figure 1 shows the results of the correspondence analysis, which maps the relationship of consumer perceptions of naturalness in terms of ingredients and countries. These two dimensions explained 67.8% of the variability, with 53.5% in the first dimension and 14.2% in the second. Groupings from cluster analysis are also indicated as ellipses that overlay the map. Cluster 1 consisted of Australia, India, Japan, South Africa, the UK, and the USA. Cluster 2 contained only Russia, and Cluster 3 comprised Brazil, China, Mexico, Peru, and Spain. Cluster 4 included only Thailand.

The perception of each cluster can be explained in high–low terms, considering a frequency higher than 50% to be high. Countries in Cluster 1 generally considered corn, wheat flour, soybean, black beans, and salt as natural. Most consumers in South Africa also consider canola oil to be natural. In Cluster 2, wheat flour, corn, salt, sugar, and pea flour were considered natural ingredients by most consumers. Interestingly, sodium bicarbonate (46.3%) and baking soda (7.8%) differed considerably in terms of consumer perceptions of naturalness. In Cluster 3, corn, soybean, pea, and wheat flours were commonly evaluated as having high naturalness; whereas, sorghum flour, salt, and maltodextrins were recognized as natural in China, Mexico, Spain, and Peru, respectively. Wheat flour from Brazil and pea flour from Spain were considered natural ingredients by fewer consumers. In Cluster 4, salt, soybean, pea flour, wheat flour, maltodextrins, and sorghum flour were recognized as natural by a high percentage of consumers, and the frequencies of people in that cluster selecting these as natural were generally higher than those in other countries.

### 3.4. Consumer Perceptions of 20 Ingredients According to Demographics

The differences in how participants from each country perceived naturalness of the 20 ingredients were analyzed according to the following demographic characteristics: age, education, sex, number of adults in the household, and number of children in the household. The results are presented in Table 4, Table 5, Table 6, Table 7 and Table 8.

#### 3.4.1. Analysis by Age Group

In the comparison of age groups (Table 4), five countries (Australia, the UK, the USA, China, and Mexico) showed significant perception differences in more than 50% of the ingredients across age groups. Typically, when perception differences were found across ingredients, older consumers were more likely than younger consumers to say that those ingredients were natural. Only China had more than one perception difference in corn, maltodextrins, and sorghum flour, where a higher percentage of younger consumers than older consumers said that the ingredients were natural. BHA and insect powder showed no differences in the percentage of consumers who perceived them as natural in any country. Notably, in both cases, the percentages were low for all countries.

#### 3.4.2. Analysis by Education Level

Among all the countries, significant perception differences were observed between the education groups for approximately 35% of the ingredients. Specifically, ingredients were perceived more frequently as natural by consumers with a higher education level in Australia (xanthan gum), Japan (molasses, sorghum flour, and soybean), South Africa (maltodextrins and soybean), the UK (canola oil and insect powder), the USA (insect powder and sorghum flour), Russia (lecithin), Brazil (sodium bicarbonate and sugar), China (corn syrup, maltodextrins, pea flour, soybean, wheat flour, and xanthan gum), Peru (canola oil, corn, and soybean), Spain (gluten, lecithin, and sorghum flour), and Thailand (baking soda, pea flour, salt, and soybean).

Only India (BHA, lecithin, SAPP, and sodium bicarbonate) and South Africa (baking soda and sugar) had more than one ingredient that was perceived as natural by more consumers with lower education levels.

#### 3.4.3. Analysis by Sex

In the comparison of the sex groups (Table 6), some differences in the perception of the various ingredients as natural were observed. Only two ingredients (sorghum and wheat flours) showed significant (*p* < 0.05) perception differences between males and females in more than four countries. In both cases, six countries showed that males were more likely than females to say that the ingredients were natural. For the other four ingredients (lecithin, salt, SAPP, and sugar), three or more countries showed perception differences between males and females. Lecithin had four countries with perception differences, with three countries having more females and one country having more males that perceived the ingredient as natural. Sugar had four countries showing differences in perceptions of naturalness between sexes, and more males in three countries and more females in one country perceived sugar as natural. Salt and SAPP showed perception differences between sexes in three countries, but salt was considered natural by more males, and SAPP was considered natural by more females.

#### 3.4.4. Analysis by Number of Adults in the Household

In the comparison of the two groups categorized by the number of adults in the household (Table 7), it was hypothesized that a higher number of adults in a household would be associated with a stronger recognition of natural ingredients compared to households with fewer adults. This hypothesis was supported only in India and China, where statistically significant perception differences were found between the groups for most ingredients. Notably, these two countries showed contrasting tendencies in the perception of ingredients as natural. For example, in India, the group with one to two adults recognized black beans as natural at a significantly higher rate; whereas, in China, the group with three or more adults showed a significantly higher recognition of black beans as natural. This trend was observed for nearly all the ingredients evaluated. In addition, although the UK, South Africa, and Australia showed significant perception differences for a few ingredients, only one or no ingredients showed such differences in the remaining eight countries.

#### 3.4.5. Analysis by Number of Children in the Household

The presence or absence of children under the age of 18 in the household (Table 8) was compared to determine whether households with children were more likely to consider factors such as health and nutrition when evaluating food ingredients. For example, the study investigated whether consumers with children were more likely to penalize ingredients and not identify them as natural because of potential health concerns. Baking soda and BHA showed no perception differences among the two groups in any country. Australia was the only country in which significant perception differences were observed between groups for most ingredients. Interestingly, in every case in Australia where significant perception differences were observed, those with no children were more likely to identify ingredients as natural. The same finding (no children in the household gave higher natural responses) occurred in the other two countries (South Africa and the UK), where there were more than three significant perception differences. Only China had more than one significant perception difference between the no-child and with-children households, where those with children were more likely to perceive some ingredients as natural (maltodextrins, sorghum flour, and soybean). Furthermore, countries where perceptions of two or fewer ingredients were found to differ between those with and without children were also of interest. Three countries had no significant perception differences (Japan, Mexico, and Peru); Russia and Spain had only one perception difference; and India, Brazil, and Thailand had two perception differences for ingredients between those with and without children in the household. Thus, in only a few countries was there much evidence of a difference in the perception of naturalness when there were no children in the household versus when children were present.

## 4. Discussion

Shared perceptions of natural ingredients recognized by consumers were observed in each country (Table 2 and Table 3 and Figure 1); however, notable perception differences also emerged. Some variations in the tendency for significant perception differences across these factors were based on demographic factors, such as age, education level, sex, and the numbers of adults and children in the household (Table 4, Table 5, Table 6, Table 7 and Table 8). However, many ingredients showed significant perception differences based on age, whereas the differences related to the number of adults and children in the household exhibited contrasting trends. Therefore, in this study, the following trends are discussed: (1) ingredients perceived as natural by nearly all countries (corn, wheat flour, and soybean); (2) ingredients generally considered natural but not in certain countries (black beans, canola oil, pea flour, salt, and sugar); (3) ingredients rarely perceived as natural (BHA and SAPP); and (4) ingredients perceived as natural in limited countries. Differences in demographic information are also discussed.

### 4.1. Overall Consumer Perceptions

#### 4.1.1. Natural Ingredients: Corn, Soybean, and Wheat Flour

As mentioned previously, the three primary ingredients (corn, wheat flour, and soybeans) are widely recognized as natural ingredients and are actively produced, consumed, imported, and exported in most countries. However, various factors, such as cultivation technologies, policies, GMO cultivation practices [23,24], and consumption behaviors, may influence consumer perceptions of these ingredients in different ways across countries. Furthermore, the relationship between production and consumption is often disproportionate. This suggests that these factors may have a complex and nuanced effect on how consumers perceive ingredients as natural.

In Thailand, only 3.2% of consumers considered corn a natural ingredient, a perception unique to the country. Therefore, it is important to consider the potential influence of cultural factors. Thai consumers rarely eat corn-based products compared to consumers in many other countries. Corn production in Thailand is less than one-fifth that in Mexico [25]. The processed sweet corn industry has received more attention in Thailand’s domestic market than in the past [26]. This industry primarily produces various products, including cornstarch and grilled sweet corn.

Wheat flour is widely regarded as a natural ingredient in many countries. Wheat consumption is expected to rise steadily due to population growth and increased middle-class income [27]. However, it was perceived as natural by less than 50% of consumers in Brazil despite the country’s significant consumption of bread, biscuits, and pasta. The Wheat Industry Association (Abitrigo) of Brazil highlights the urgent need for quality improvement, as only approximately 30% of Brazilian wheat flour meets the standards required for bread production, owing to its poor quality [27]. This quality problem may influence Brazilians’ belief that corn is natural.

#### 4.1.2. Ingredients Generally Considered as Natural: Black Beans, Canola Oil, Pea Flour, Salt, and Sugar

Black beans are classified as NOVA Group 1 (minimally processed foods); and canola oil, pea flour, salt, and sugar are classified as NOVA Group 2 (processed culinary ingredients [28]. They were considered natural by more than 40% of the consumers in at least seven countries.

Black beans have been a staple food in the North American diet for at least 7000 years. Known as ‘turtle beans’ in English and ‘frijoles negros’ in Spanish, they are recognized as a valuable source of essential nutrients that offer various health benefits, including blood sugar regulation, cancer prevention, eye and heart health, and weight management [29]. The global production of black beans is primarily concentrated in Brazil, Mexico, and Argentina, with the USA also contributing substantially to the international market and Brazil being the largest consumer of black beans [30]. However, in this study, only 8.1% of consumers in Brazil perceived black beans as natural. Thai consumers did not consider black beans natural. However, consumers in Brazil and Thailand may not have understood the question. The Portuguese translation of black beans is quite similar to that of black bean stew, a common food in Brazil. Therefore, it is possible that the respondents confused the term with black bean stew and did not consider it an ingredient. Similarly, in Thailand, the term black bean has multiple translations, depending on the type of black bean and use. Hence, the translation and use of only one term may have confused consumers, resulting in a low percentage of consumers saying that black beans are natural. Furthermore, Rotjanapaiboon [31] indicated that there is generally a high acceptance of dishes made with dried black beans in Thailand. Moreover, studies on consumer patterns and perceptions of black beans in Thailand are relatively limited. Although the perception is generally perceived as positive, further exploring the perceptions of Thai consumers of black beans based on the findings of this study will be valuable.

The global pea flour market is estimated to reach USD 22.2 billion in 2023, with a projected Compound Annual Growth Rate of 14.5% from 2023 to 2030 [32]. Pea flour is widely recognized as a popular ingredient in the food industry, owing to its extensive use in baking, cooking, and as a protein supplement. Increasing consumer demand for plant-based and gluten-free products has contributed considerably to the growth of the pea flour market. Because consumers actively seek alternatives to animal-based protein sources, they are likely to be attracted to the nutritional benefits of pea flour. This trend is further supported by an increase in clean-label products that emphasize minimal processing and easily recognizable ingredients [32].

Unexpectedly, the overall proportion of consumers perceiving salt and sugar as natural varied across countries. Chambers [33] showed that more than 70% of respondents in a study of 30 ingredients answered that sea salt was natural. In this study, it was presented as salt and consumers may have thought of iodized salt or reduced-sodium salts available in countries with high risk of iodine deficiency [34] or cardiovascular diseases [35], respectively.

Additionally, the results of Chambers [33] for sugar should be compared with those of this study. Chambers [33] found that sugar was generally perceived as natural with a few consumers responding that it is ‘probably from a factory (5.1%)’, ‘an artificial ingredient (6.0%)’, or ‘artificial chemical used in processing (7.8%)’.

Canola oil is a unique type of rapeseed oil. Global production data from the United States Department of Agriculture [36] showed that four countries/regions produce the most rapeseed oil (European Union: 30%, China: 22%, Canada: 14%, and India: 12%). In most countries, the perception of canola oil was moderate, perhaps because it is generally used in North America and Australia, with many other countries referring to it as rapeseed oil. In both Russia and Spain, canola oil had a low percentage of consumers who considered it natural. This may be partly due to a terminology issue. However, this may also be associated with distrust in Russia. Russian consumers have a widespread perception that imported products with long expiration dates incorporate large amounts of additives [37]. Other authors have shown that foods with high import and export may be associated with some level of distrust related to aspects, such as organic status, which is associated with being natural by some [38]. However, South Africa’s canola oil has been found to be safe for human consumption, of high quality, and more cost-effective than olive oil [39]. Thus, this consumer-friendly information may have contributed to the highest rate (61%) of natural perceptions among all the countries.

#### 4.1.3. Ingredients Rarely Perceived as Natural: BHA and SAPP

BHA and SAPP are classified as NOVA Group 4, and any foods containing these ingredients would be classified as ultra-processed foods [28]. These ingredients were not expected to be perceived as natural by most consumers. Accordingly, consumers rarely considered them natural, with the highest percentage (4.0%) found in India (Table 3). Therefore, perceptions of these ingredients are commonly unaffected by country differences. According to a study of consumers in the United States, BHA was the ingredient with the lowest percentage of consumers recognizing it as natural, with approximately 60% of respondents answering the question ‘Do not know what it is [33]’. Similarly, SAPP, an ingredient in baking powder, has a chemical-sounding name and is a chemically manufactured product. As most consumers in developed countries want natural foods free from synthetic ingredients [9], it is important to educate consumers about the roles of BHA and SAPP.

#### 4.1.4. Mixed Natural Ingredient Perceptions

In addition to the previously mentioned ingredients, a pronounced proportion of the remaining 10 ingredients were considered natural only in specific countries. Notably, corn syrup was perceived as more natural than corn. Furthermore, differences in the perception of baking soda and sodium bicarbonate, which are different names for the same product, were observed. These differences reflect the ambiguous boundaries of what consumers perceive as natural.

Respondents in Peru (70.6%) and Thailand (65.1%) considered maltodextrins as a natural ingredient at a high percentage compared with respondents in other countries. Both of these countries tax sugar-sweetened beverages and, in both countries, the use of maltodextrins is widespread as an addition to beverages to provide some sweetness and thickness to beverages. They can also be made from locally sourced materials (rice in Thailand and corn and potatoes in Peru). Thus, it may be possible that these countries recognize the ingredient more than respondents in other countries that may perceive the term as chemical-sounding. However, it is unclear why this is not true in other countries with sugar taxes or the widespread use of maltodextrins. Maltodextrins are ingredients that the FDA [40] generally recognizes as safe and are widely used as an affordable additive because they share similar properties with sugar [41].

According to the United States Department of Agriculture [42], global sorghum production has reached 61 million tons, with the United States accounting for 19% of the total production. Sorghum is primarily grown in Asia, which hosts some of the main sorghum-producing countries. More than 80% of the global sorghum production is concentrated in Africa (61%) and Asia (22%) [43,44]. In Asia, sorghum cultivation is concentrated in India and China, where it is used to produce roti (flatbread), porridge, starch, and alcoholic beverages [44,45]. Sorghum is cultivated across Thailand, with the central and northeastern regions being the most prominent areas of growth. Although a substantial portion of the sorghum produced is exported, some is used domestically for animal feed and ethanol production [46,47,48,49]. Given this context, it is believed that the perception of sorghum flour as a natural ingredient may be more pronounced in Thailand, China, and South Africa compared with that in the other countries. This is likely because of the country’s self-sufficiency in sorghum production and its widespread use of foods commonly consumed by consumers.

Corn syrup is an ingredient made from cornstarch that is partially decomposed using acids and enzymes. The ingredient was considered a natural ingredient by nearly 50% of the consumers in Thailand (48.4%), Mexico (46.3%), and Spain (44.6%). In contrast, only a small percentage of Japanese consumers (9.2%) perceived it as natural. Notably, more Thai respondents than those from other countries regarded corn syrup as natural, where the proportion of those who considered corn syrup natural was about 16 times greater than those who perceived corn as natural. This difference in perception could be due to various factors in Thailand, such as import restrictions, environmental concerns, and production practices. Thailand does not allow the production of genetically modified (GM) crops for commercial use, but imports GM crops, which may have contributed to the perception that corn is less natural [50,51] than corn syrup.

In 2021, the global market for high-fructose corn syrup (HFCS) was estimated to be approximately USD 9.1 billion. Furthermore, Market Research Report [52] projects that this figure will reach around USD 10.6 billion by 2030. Notably, North America and Europe produce the highest volumes of products containing corn-syrup-based sweeteners. The report [52] also indicated that owing to its distinct characteristics, HFCS has broader applications in processed and convenient foods than cane sugar, leading to an increase in consumer preference. Despite the increasing inclusion of HFCS in various packaged foods over the past decade, consumers tend to avoid packaged goods labeled with HFCS or, the newer term, corn sugar [52]. Additionally, Chambers [33] found that only 11.3% of respondents were unfamiliar with the term HFCS as either a product or ingredient. Approximately 27% of consumers associated the term with artificial ingredients, whereas nearly 17% described it using terms such as processed, artificial, or chemical ingredient and expressed concerns that it ‘sounds dangerous’ or ‘bad for health [33]’. In comparison, fewer than 5% of consumers expressed such views regarding corn itself, thus suggesting a more favorable perception of corn compared to HFCS.

Several contrasting results between baking soda and sodium bicarbonate were found. Russia, which perceived the chemical name of baking soda (sodium bicarbonate; 46.4%) as natural, did not recognize baking soda (7.8%) as natural. Before the collapse of the Soviet Union, the term sodium bicarbonate was used on packaging, and since then, both soda for baking and sodium bicarbonate are used interchangeably [53]. Most participants in this study were most likely accustomed to the term sodium bicarbonate and not baking soda, which explains this difference. In some other countries, it is commonly used in ingredient lists as bicarbonate of soda. In the USA, linguistic familiarity with baking soda is generally high, and the result was 33.2% higher than that of sodium bicarbonate (13.0%), which is the highest level among the 13 countries. The types of products available in the United States vary slightly. Although both ingredients are listed in the food product ingredient lists, there is a tendency to differentiate between them based on the type of product. In contrast, consumers in Japan and Thailand were the only ones where respondents tended not to consider either of the two ingredients natural.

In general, the proportion of consumers who consider insect powder a natural ingredient was low. However, in Mexico, Russia, and Japan, a slightly higher percentage of consumers perceived it as natural than in other countries. Furthermore, Castro [17] found that more than 60% of respondents in these countries expressed a willingness to consume insect powder as a familiar product. Although the findings of this study indicated that consumers did not consider insect powder a natural ingredient, positive responses regarding the intention to consume insect powder have also been observed in other countries [20,54]. This phenomenon is believed to be influenced by factors such as unfamiliarity, consumer beliefs, and culture of entomophagy [17,55,56]. In Mexico, over 500 species of insects are used as food ingredients, often enjoyed as appetizers, or incorporated into meals through various recipes [54]. Insect-based products are recognized and consumed locally [21].

The perception of gluten, a constituent and a byproduct of wheat flour [33], generally was not perceived as natural and certainly not at the level of wheat flour. A prior study in the USA found that gluten was not considered natural, although it was perceived as healthy, but not organic. However, more than 25% of the consumers in Australia, China, and the UK perceive gluten as natural. This result could be caused by the lack of emphasis on gluten intolerance in social media in those countries. Notably, in Thailand, Russia, and India, the percentage of consumers considering wheat flour as natural was seven to ten times higher, a much wider gap than that in other countries.

In a similar vein, ingredients such as molasses, xanthan gum, and lecithin were unfamiliar to nearly 60% of respondents, and they were also considered as a chemical or artificial ingredient by consumers [33]. Furthermore, given that lecithin exists in various forms, such as egg lecithin, soy lecithin, and sunflower lecithin, it is likely that perception for a natural ingredient will differ across countries depending on which type of lecithin is commonly used in each country.

#### 4.1.5. Demographic Characteristics

Older individuals were expected to have more background knowledge of food and ingredients. This held true for most countries, including those with the largest differences in ingredients by age group (Australia, the UK, the USA, China, and Mexico). Among the 72 studies reviewed by Román [9] on the importance of food naturalness, a significantly higher proportion of older adult participants considered it important [57,58,59,60,61,62].

The mixed results for the differences in education levels were not surprising. Previous studies have explored the effects of educational level on food choice and naturalness. Two studies that included educational levels showed unclear results, and one study showed no differences due to education level [63]. Another study [64] found that of those groups most interested in naturalness, two had higher levels of education and one did not. This suggests that although education level might be important, it does not seem to be a primary influence.

Román [9] found that women were significantly more likely to consider food naturalness important. This study did not show notable sex differences, similar to previous studies [57,65]. The importance of naturalness to women may not translate into differences in the likelihood that women will perceive specific ingredients as more natural.

In China, the health and wellness of the food market has been continuously growing due to increased interest in health driven by economic growth and an aging population. In particular, the emergence and rapid rise in Green Food can be attributed to Chinese consumers’ growing concerns and diminished trust in domestic food products, especially after food safety incidents, such as the melamine milk scandal [66,67]. Thus, both the national focus on green foods and organic food industries along with heightened consumer interest in health-related dietary habits have likely influenced these shifts in consumer behavior [68].

The study findings suggest that the presence of children may influence perceptions, possibly because of heightened awareness and caution regarding food choices and consumption. For example, one study [69] found that children and adolescents are generally more taste-oriented in their food choice, and they are influenced by factors other than food preferences to a greater extent than adults. Thus, adults often assume the role of gatekeepers and may be more selective toward foods containing natural ingredients when children are present. Notably, the demographic effect of children in the household was not universal, with countries such as Japan, Mexico, and Peru showing no significant effects of having children in the household on the perception of ingredients as natural. Cultural differences are likely to explain some of these differences. In countries such as Japan, where processed foods constitute only 25% of a child’s caloric intake [70], the impact of ingredients may be less important.

### 4.2. Limitations

A key limitation of this study is that only ingredient terms were listed and no additional information on why consumers thought those ingredients were natural was obtained. Although the study attempted to make comparisons with prior research on the perception of natural ingredients and eating patterns, the study included questions related to familiarity or other issues, such as those in prior studies [33], which would have allowed additional analyses.

In addition, the data collection was based exclusively on the CATA method. This approach does not provide in-depth insights into why the participants selected certain ingredients or how they defined and perceived the term natural. Consequently, interpreting and standardizing findings in a specific direction can be challenging. Therefore, to better understand the reasoning and perceptions of participants, future research should aim to gather additional information [33,71] that complements CATA data, such as surveys [33,72,73,74,75] or eye-tracking [76,77,78,79,80]. In addition, the CATA method may underestimate the actual percentages [81,82], and when foods are perceived as natural, the CATA method does not allow for understanding of the levels of variables that other methods may allow [83].

As with surveys conducted in multiple languages, translation and adaptation issues may arise. It is possible that the terms used in English do not translate well into other languages and can cause misunderstanding by consumers. Although an accepted method for translation was used, the terms may have not completely aligned among the languages. In addition, although online surveys are common, there may be bias, especially when testing internationally, with lower participation by those without easy access to technology.

## 5. Conclusions

Countries have different perceptions of which ingredients are natural. Demographics such as age, sex, and, to a lesser extent, number of adults or children in the household impact those differences depending on which country is being studied. Based on these results and the extant literature, consumer perceptions appear to be influenced by familiarity and various geographic and demographic factors. Furthermore, consumers lack awareness of what natural is. This study shows that the term natural is clearly interpreted differently when it comes to various food ingredients. Thus, creating a single universally accepted definition of ‘natural’ is challenging and probably impossible. This study provides information that brings us closer to fully understanding the differences in ingredients and perceptions of naturalness globally. By obtaining insights into the perceptions of consumers from both WEIRD and non-WEIRD countries, this research contributes to the transformation of the food industry with a focus on the differences among countries and demographics.

The insights gained from this research provide valuable data for improving marketing strategies and encouraging better food choices for consumers in the natural product sector.

## Figures and Tables

**Table 1:** Consumer demographic percentages *.

Demographic Characteristics	Variables	Percentage
Age	18–34	33%
	35–54	33%
	55+	33%
Education Level	Primary School or Less	2%
	High School	25%
	College or University	73%
Sex	Male	50%
	Female	50%
Number of Adults Living at Residence	1	10%
	2	32%
	3	27%
	4	16%
	5+	14%
Number of Children Living at Residence	0	54%
	1	26%
	2	15%
	3+	5%

* Percentages were calculated based on a total of 8191 consumers.

**Table 2:** Percentage of consumers who perceived specific ingredients as natural.

Ingredient	Frequency of Consumers Perceiving as Natural	Percent (%)
Corn	5988	73.10
Wheat flour	5715	69.77
Soybean	5656	69.05
Pea flour	4814	58.77
Salt	4687	57.22
Black beans	3354	40.95
Sugar	3338	40.75
Canola oil	2821	34.44
Sorghum flour	2534	30.94
Corn syrup	2368	28.91
Maltodextrins	1887	23.04
Molasses	1620	19.78
Gluten	1513	18.47
Baking soda	1232	15.04
Sodium bicarbonate	1186	14.48
Xanthan gum	964	11.77
Insect powder	893	10.90
Lecithin	712	8.69
SAPP (Sodium acid pyrophosphate)	167	2.04
BHA (Butylated hydroxyanisole)	117	1.43
Total	8191	100.00

**Table 3:** Cochran’s Q test of the frequency that 20 ingredients are perceived as natural in 13 countries.

	Australia	Brazil	China	India	Japan	Mexico	Peru	Russia	South Africa	Spain	Thailand	United Kingdom	USA	*p*-Value
Baking soda	21.0 ^b^ *	18.1 ^bc^	16.3 ^bc^	14.9 ^bc^	1.7 ^e^	18.4 ^bc^	6.3 ^de^	7.8 ^de^	12.9 ^cd^	18.4 ^bc^	5.6 ^e^	21.0 ^b^	33.2 ^a^	<0.0001
BHA	2.2 ^abc^	1.7 ^abc^	1.1 ^bc^	3.0 ^ab^	0.2 ^c^	1.4 ^bc^	0.6 ^c^	0.2 ^c^	1.0 ^bc^	0.6 ^c^	1.1 ^bc^	1.6 ^abc^	3.8 ^a^	<0.0001
Black beans	61.4 ^ab^	8.1 ^ef^	12.2 ^e^	54.6 ^b^	62.5 ^ab^	24.1 ^d^	10.3 ^ef^	65.2 ^a^	68.1 ^a^	36.0 ^c^	1.1 ^f^	65.7 ^a^	62.7 ^ab^	<0.0001
Canola oil	43.3 ^cd^	45.1 ^bc^	52.9 ^ab^	34.3 ^de^	27.9 ^ef^	40.6 ^cd^	30.2 ^ef^	9.7 ^g^	61.0 ^a^	11.1 ^g^	22.2 ^f^	27.6 ^ef^	41.9 ^cd^	<0.0001
Corn	79.5 ^cde^	83.3 ^bcd^	74.8 ^def^	82.9 ^bcd^	61.6 ^g^	92.2 ^a^	89.5 ^ab^	84.9 ^abc^	77.0 ^cdef^	80.2 ^cde^	3.2 ^h^	72.2 ^ef^	69.0 ^fg^	<0.0001
Corn syrup	22.1 ^c^	22.5 ^c^	27.8 ^bc^	21.9 ^c^	9.2 ^d^	46.3 ^a^	32.9 ^b^	27.0 ^bc^	26.3 ^bc^	44.6 ^a^	48.4 ^a^	27.3 ^bc^	19.5 ^c^	<0.0001
Gluten	26.2 ^ab^	11.6 ^ef^	28.3 ^ab^	12.7 ^ef^	13.5 ^ef^	14.4 ^def^	16.0 ^cde^	10.0 ^ef^	21.3 ^bcd^	24.1 ^b^	7.3 ^f^	31.7 ^a^	23.0 ^bc^	<0.0001
Insect powder	4.8 ^f^	12.2 ^bcde^	14.1 ^bcd^	4.4 ^f^	15.9 ^bc^	27.8 ^a^	7.1 ^ef^	18.1 ^b^	3.7 ^f^	10.8 ^cde^	8.6 ^def^	7.1 ^ef^	7.1 ^ef^	<0.0001
Lecithin	21.4 ^a^	4.8 ^cd^	7.5 ^c^	5.9 ^cd^	5.9 ^cd^	3.8 ^cd^	0.8 ^d^	15.2 ^b^	15.9 ^b^	4.3 ^cd^	1.0 ^d^	13.0 ^b^	13.7 ^b^	<0.0001
Maltodextrins	6.5 ^e^	38.9 ^bc^	42.2 ^b^	3.8 ^e^	1.4 ^e^	21.1 ^d^	70.6 ^a^	2.7 ^e^	3.0 ^e^	34.0 ^c^	65.1 ^a^	5.6 ^e^	4.6 ^e^	<0.0001
Molasses	39.4 ^ab^	3.2 ^de^	9.5 ^d^	23.8 ^c^	36.8 ^b^	1.9 ^de^	2.1 ^de^	8.1 ^de^	44.6 ^a^	2.1 ^de^	1.1 ^e^	39.5 ^ab^	45.1 ^a^	<0.0001
Pea flour	38.4 ^fg^	77.5 ^bc^	66.7 ^d^	49.5 ^e^	30.0 ^g^	86.7 ^ab^	80.3 ^ab^	69.2 ^cd^	50.2 ^e^	47.3 ^ef^	88.3 ^a^	41.9 ^ef^	38.1 ^fg^	<0.0001
Salt	57.9 ^cd^	35.2 ^f^	33.0 ^f^	62.2 ^c^	49.8 ^de^	57.0 ^cde^	48.3 ^e^	78.1 ^b^	57.0 ^cde^	62.7 ^c^	93.7 ^a^	54.4 ^cde^	54.4 ^cde^	<0.0001
SAPP	2.7 ^ab^	2.4 ^ab^	2.2 ^ab^	4.0 ^a^	1.3 ^ab^	1.6 ^ab^	0.6 ^b^	1.3 ^ab^	2.2 ^ab^	1.1 ^b^	1.6 ^ab^	2.4 ^ab^	3.2 ^ab^	0.001
Sodium bicarbonate	19.5 ^bc^	10.5 ^def^	6.2 ^ef^	6.0 ^ef^	3.8 ^f^	25.1 ^b^	13.2 ^cd^	46.3 ^a^	11.0 ^de^	13.3 ^cd^	3.8 ^f^	16.5 ^cd^	13.0 ^cd^	<0.0001
Sorghum flour	36.3 ^c^	18.3 ^fg^	60.3 ^a^	26.7 ^def^	2.1 ^h^	35.4 ^cd^	22.4 ^fg^	32.1 ^cde^	45.4 ^b^	20.8 ^fg^	63.3 ^a^	15.1 ^g^	24.0 ^efg^	<0.0001
Soybean	68.9 ^cd^	81.1 ^b^	67.0 ^cde^	81.7 ^b^	71.6 ^c^	61.9 ^de^	69.7 ^cd^	41.3 ^f^	69.8 ^cd^	70.0 ^cd^	94.1 ^a^	58.9 ^e^	61.6 ^de^	<0.0001
Sugar	50.3 ^bcd^	41.3 ^de^	20.8 ^g^	50.8 ^bc^	45.1 ^bcd^	32.5 ^ef^	32.5 ^ef^	72.5 ^a^	41.9 ^cde^	24.6 ^fg^	17.1 ^g^	46.3 ^bcd^	53.8 ^b^	<0.0001
Wheat flour	65.7 ^cde^	42.5 ^f^	68.7 ^bcde^	86.0 ^a^	61.3 ^e^	64.9 ^de^	75.9 ^b^	91.9 ^a^	71.9 ^bcd^	73.8 ^bc^	73.2 ^bcd^	68.3 ^bcde^	62.9 ^e^	<0.0001
Xanthan gum	8.9 ^ef^	16.3 ^bc^	15.4 ^bcd^	4.8 ^fg^	1.4 ^g^	31.1 ^a^	21.3 ^b^	4.8 ^fg^	7.8 ^ef^	13.0 ^cde^	9.7 ^def^	10.3 ^cdef^	8.3 ^ef^	<0.0001

* Different letters indicate statistically significant differences between countries based on pairwise comparisons.

**Table 4:** Influence of age on the perception of 20 ingredients as natural.

Ingredient	Age	Cluster 1						Cluster 2	Cluster 3					Cluster 4
		Australia	India	Japan	South Africa	United Kingdom	USA	Russia	Brazil	China	Mexico	Peru	Spain	Thailand
Baking soda	18–34 yrs	**21.0 ^ab^**	**20.4 ^a^**	1.9	10.5	16.7	**26.3 ^b^**	4.8	**7.1 ^b^**	**9.5 ^b^**	**8.1 ^c^**	**2.4 ^b^**	**8.6 ^b^**	4.8
	35–54 yrs	**15.7 ^b^**	**13.3 ^b^**	2.4	13.8	22.9	**33.2 ^ab^**	11.0	**20.5 ^a^**	**22.4 ^a^**	**18.6 ^b^**	**6.2 ^ab^**	**21.4 ^a^**	3.3
	55 yrs +	**26.2 ^a^**	**11.0 ^b^**	1.0	14.3	23.3	**40.0 ^a^**	7.6	**26.7 ^a^**	**17.1 ^a^**	**28.6 ^a^**	**10.5 ^a^**	**25.2 ^a^**	8.6
	*p*-value	**0.031**	**0.019**	0.524	0.447	0.174	**0.012**	0.060	**<0.0001**	**0.002**	**<0.0001**	**0.003**	**<0.0001**	0.053
BHA	18–34 yrs	2.4	5.2	0.0	1.9	1.0	5.3	0.5	2.4	0.5	1.4	0.5	0.5	2.4
	35–54 yrs	2.4	1.9	0.5	1.0	2.4	2.4	0.0	0.5	1.9	1.4	0.5	0.0	0.5
	55 yrs +	1.9	1.9	0.0	0.0	1.4	3.8	0.0	2.4	1.0	1.4	1.0	1.4	0.5
	*p*-value	0.930	0.072	0.368	0.133	0.492	0.302	0.368	0.228	0.364	1.000	0.778	0.172	0.099
Black beans	18–34 yrs	**51.9 ^b^**	53.6	**50.0 ^c^**	**61.9 ^b^**	**57.1 ^b^**	**50.2 ^b^**	63.3	7.1	11.9	**21.0 ^b^**	9.5	30.5	1.4
	35–54 yrs	**58.1 ^b^**	55.7	**61.4 ^b^**	**65.7 ^b^**	**70.0 ^a^**	**66.4 ^a^**	68.6	6.2	10.5	**19.5 ^b^**	10.0	36.7	0.0
	55 yrs +	**74.3 ^a^**	54.8	**76.2 ^a^**	**76.7 ^a^**	**70.0 ^a^**	**71.4 ^a^**	63.8	11.0	14.3	**31.9 ^a^**	11.4	41.0	1.9
	*p*-value	**<0.0001**	0.905	**<0.0001**	**0.003**	**0.006**	**<0.0001**	0.460	0.167	0.485	**0.005**	0.800	0.080	0.153
Canola oil	18–34 yrs	**32.9 ^b^**	**30.3 ^b^**	27.6	**52.9 ^b^**	24.3	**33.5 ^b^**	12.9	44.8	52.4	38.1	29.5	11.9	**12.4 ^b^**
	35–54 yrs	**39.0 ^b^**	**29.5 ^b^**	27.6	**61.9 ^ab^**	32.9	**43.6 ^a^**	9.5	42.9	59.0	39.5	29.0	11.0	**23.8 ^a^**
	55 yrs +	**58.1 ^a^**	**42.9 ^a^**	28.6	**68.1 ^a^**	25.7	**48.6 ^a^**	6.7	47.6	47.1	44.3	31.9	10.5	**30.5 ^a^**
	*p*-value	**<0.0001**	**0.005**	0.969	**0.006**	0.110	**0.006**	0.100	0.615	0.050	0.401	0.792	0.894	**<0.0001**
Corn	18–34 yrs	**71.0 ^c^**	85.8	**51.9 ^b^**	80.0	**61.4 ^b^**	65.6	82.9	81.0	**78.6 ^a^**	91.4	**84.8 ^b^**	76.2	4.8
	35–54 yrs	**79.5 ^b^**	81.0	**61.9 ^a^**	72.9	**80.5 ^a^**	70.6	86.7	85.2	**81.0 ^a^**	90.0	**91.4 ^a^**	80.0	1.9
	55 yrs +	**88.1 ^a^**	81.9	**71.0 ^a^**	78.1	**74.8 ^a^**	71.0	85.2	83.8	**64.8 ^b^**	95.2	**92.4 ^a^**	84.3	2.9
	*p*-value	**<0.0001**	0.379	**0.000**	0.198	**<0.0001**	0.408	0.545	0.487	**0.000**	0.117	**0.021**	0.115	0.236
Corn syrup	18–34 yrs	**15.7 ^b^**	21.8	9.0	**21.4 ^b^**	**18.6 ^b^**	**13.4 ^b^**	26.2	25.2	**28.6 ^ab^**	**41.4 ^b^**	38.6	**33.8 ^b^**	50.5
	35–54 yrs	**16.7 ^b^**	24.8	8.6	**22.9 ^b^**	**26.7 ^b^**	**21.3 ^a^**	30.0	18.6	**33.8 ^a^**	**44.3 ^ab^**	27.6	**40.0 ^b^**	52.4
	55 yrs +	**33.8 ^a^**	19.0	10.0	**34.8 ^a^**	**36.7 ^a^**	**23.8 ^a^**	24.8	23.8	**21.0 ^b^**	**53.3 ^a^**	32.4	**60.0 ^a^**	42.4
	*p*-value	**<0.0001**	0.367	0.876	**0.003**	**0.000**	**0.020**	0.458	0.228	**0.013**	**0.039**	0.057	**<0.0001**	0.094
Gluten	18–34 yrs	**23.8 ^b^**	13.7	**10.0 ^b^**	19.0	30.5	26.8	10.0	**8.1 ^b^**	**25.2 ^b^**	**16.2 ^a^**	**10.5 ^b^**	21.9	9.5
	35–54 yrs	**18.1 ^b^**	14.8	**18.6 ^a^**	19.0	31.9	21.8	11.9	**11.0 ^ab^**	**38.1 ^a^**	**9.0 ^b^**	**14.3 ^b^**	21.4	3.8
	55 yrs +	**36.7 ^a^**	9.5	**11.9 ^b^**	25.7	32.9	20.5	8.1	**15.7 ^a^**	**21.4 ^b^**	**18.1 ^a^**	**23.3 ^a^**	29.0	8.6
	*p*-value	**<0.0001**	0.232	**0.026**	0.156	0.870	0.270	0.429	**0.048**	**0.000**	**0.021**	**0.001**	0.124	0.055
Insect powder	18–34 yrs	5.2	6.2	17.1	5.7	9.0	7.7	20.5	12.4	14.8	25.7	7.1	7.1	8.6
	35–54 yrs	6.7	2.9	13.3	2.9	7.6	7.1	18.1	12.4	11.9	28.1	6.2	13.3	7.6
	55 yrs +	2.4	4.3	17.1	2.4	4.8	6.7	15.7	11.9	15.7	29.5	8.1	11.9	9.5
	*p*-value	0.111	0.256	0.468	0.144	0.222	0.926	0.448	0.985	0.507	0.679	0.751	0.101	0.785
Lecithin	18–34 yrs	**9.5 ^b^**	6.2	3.8	**4.8 ^c^**	**7.1 ^b^**	**9.1 ^b^**	12.4	3.3	7.6	3.3	1.0	4.8	1.4
	35–54 yrs	**13.8 ^b^**	5.2	8.1	**12.4 ^b^**	**12.4 ^b^**	**11.4 ^b^**	18.1	5.2	4.8	2.9	0.5	3.8	0.0
	55 yrs +	**41.0 ^a^**	6.2	5.7	**30.5 ^a^**	**19.5 ^a^**	**20.5 ^a^**	15.2	5.7	10.0	5.2	1.0	4.3	1.4
	*p*-value	**<0.0001**	0.894	0.174	**<0.0001**	**0.001**	**0.002**	0.266	0.480	0.124	0.403	0.818	0.891	0.220
Maltodextrins	18–34 yrs	**9.0 ^a^**	3.3	1.4	2.9	**2.4 ^b^**	4.3	3.3	39.0	**46.2 ^a^**	**15.2 ^b^**	66.7	**27.6 ^b^**	62.9
	35–54 yrs	**2.9 ^b^**	3.3	1.4	4.8	**7.1 ^a^**	3.8	3.3	39.0	**51.4 ^a^**	**22.9 ^ab^**	71.0	**34.8 ^ab^**	64.8
	55 yrs +	**7.6 ^a^**	4.8	1.4	1.4	**7.1 ^a^**	5.7	1.4	38.6	**29.0 ^b^**	**25.2 ^a^**	74.3	**39.5 ^a^**	67.6
	*p*-value	**0.027**	0.674	1.000	0.135	**0.049**	0.623	0.381	0.993	**<0.0001**	**0.032**	0.229	**0.035**	0.589
Molasses	18–34 yrs	**24.8 ^b^**	18.5	32.4	**26.7 c**	**20.0 ^c^**	**34.4 ^c^**	7.6	4.3	11.4	2.9	1.9	3.8	1.0
	35–54 yrs	**29.0 ^b^**	25.2	35.7	**44.8 ^b^**	**42.4 ^b^**	**45.5 ^b^**	7.1	2.4	6.2	1.4	1.4	1.0	1.0
	55 yrs +	**64.3 ^a^**	27.6	42.4	**62.4 ^a^**	**56.2 ^a^**	**55.2 ^a^**	9.5	2.9	11.0	1.4	2.9	1.4	1.4
	*p*-value	**<0.0001**	0.074	0.097	**<0.0001**	**<0.0001**	**0.000**	0.639	0.512	0.130	0.466	0.578	0.088	0.866
Pea flour	18–34 yrs	**31.4 ^b^**	47.4	**21.0 ^b^**	**42.9 ^b^**	35.2	**32.1 ^b^**	62.9	74.3	**71.0 ^a^**	**82.9 ^b^**	**70.0 ^b^**	**41.4 ^b^**	84.8
	35–54 yrs	**33.8 ^b^**	47.6	**35.2 ^a^**	**48.1 ^b^**	46.2	**38.4 ^ab^**	72.4	78.1	**75.7 ^a^**	**85.2 ^b^**	**81.9 ^a^**	**45.7 ^ab^**	88.6
	55 yrs +	**50.0 ^a^**	53.8	**33.8 ^a^**	**59.5 ^a^**	44.3	**43.8 ^a^**	72.4	80.0	**53.3 ^b^**	**91.9 ^a^**	**89.0 ^a^**	**54.8 ^a^**	91.4
	*p*-value	**0.000**	0.329	**0.002**	**0.002**	0.052	**0.047**	0.051	0.362	**<0.0001**	**0.018**	**<0.0001**	**0.020**	0.104
Salt	18–34 yrs	**52.9 ^b^**	66.4	**52.9 ^a^**	54.3	**47.1 ^b^**	51.2	72.9	34.3	33.3	**48.6 ^b^**	**40.0 ^b^**	**56.2 ^b^**	91.9
	35–54 yrs	**50.5 ^b^**	61.4	**41.9 ^b^**	55.2	**56.2 ^ab^**	56.4	77.6	33.3	37.6	**56.2 ^b^**	**50.0 ^a^**	**61.0 ^b^**	95.2
	55 yrs +	**70.5 ^a^**	59.0	**54.8 ^a^**	61.4	**60.0 ^a^**	55.7	83.8	38.1	28.1	**66.2 ^a^**	**54.8 ^a^**	**71.0 ^a^**	93.8
	*p*-value	**<0.0001**	0.289	**0.018**	0.276	**0.025**	0.510	0.025	0.558	0.116	**0.001**	**0.009**	**0.006**	0.373
SAPP	18–34 yrs	2.9	**6.6 ^a^**	1.0	**4.8 ^a^**	1.0	2.9	1.4	2.4	3.8	1.9	0.5	1.4	1.9
	35–54 yrs	2.4	**2.9 ^b^**	1.9	**1.4 ^b^**	3.8	3.3	1.9	1.0	1.4	1.9	0.5	0.5	1.0
	55 yrs +	2.9	**2.4 ^b^**	1.0	**0.5 ^b^**	2.4	3.3	0.5	3.8	1.4	1.0	1.0	1.4	1.9
	*p*-value	0.941	**0.050**	0.603	**0.008**	0.159	0.954	0.413	0.159	0.161	0.666	0.778	0.562	0.666
Sodium bicarbonate	18–34 yrs	**15.2 ^b^**	8.1	3.8	**6.7 ^b^**	**11.0 ^b^**	9.6	40.5	**7.1 ^b^**	**5.2 ^ab^**	**21.4 ^b^**	**8.6 ^b^**	**8.1 ^b^**	**8.1 ^a^**
	35–54 yrs	**15.2 ^b^**	5.7	3.8	**10.5 ^ab^**	**18.6 ^a^**	12.3	46.7	**9.0 ^b^**	**3.8 ^b^**	**21.0 ^b^**	**14.3 ^ab^**	**10.5 ^b^**	**0.5 ^b^**
	55 yrs +	**28.1 ^a^**	4.3	3.8	**15.7 ^a^**	**20.0 ^a^**	17.1	51.9	**15.2 ^a^**	**9.5 ^a^**	**32.9 ^a^**	**16.7 ^a^**	**21.4 ^a^**	**2.9 ^b^**
	*p*-value	**0.001**	0.260	1.000	**0.012**	**0.027**	0.066	0.063	**0.018**	**0.041**	**0.006**	**0.042**	**0.000**	**0.000**
Sorghum flour	18–34 yrs	**22.4 ^b^**	27.0	1.4	**30.5 ^b^**	**9.0 ^b^**	**16.3 ^b^**	**23.3 ^b^**	14.3	**61.4 ^a^**	**24.8 ^b^**	18.6	**14.8 ^b^**	59.5
	35–54 yrs	**28.1 ^b^**	28.6	3.3	**49.0 ^a^**	**17.6 ^a^**	**22.7 ^b^**	**35.7 ^a^**	19.0	**70.5 ^a^**	**37.1 ^a^**	22.4	**17.6 ^b^**	62.9
	55 yrs +	**58.6 ^a^**	24.8	1.4	**56.7 ^a^**	**18.6 ^a^**	**32.9 ^a^**	**37.1 ^a^**	21.4	**49.0 ^b^**	**44.3 ^a^**	26.2	**30.0 ^a^**	67.6
	*p*-value	**<0.0001**	0.676	0.285	**<0.0001**	**0.011**	**0.000**	**0.004**	0.156	**<0.0001**	**0.000**	0.174	**0.000**	0.224
Soybean	18–34 yrs	**65.7 ^b^**	81.5	**63.8 ^b^**	66.2	**9.0 ^b^**	**52.6 ^b^**	**33.3 ^b^**	81.0	**73.3 ^a^**	**59.5 ^b^**	**59.0 ^c^**	71.0	92.4
	35–54 yrs	**61.0 ^b^**	79.5	**73.8 ^a^**	68.1	**17.6 ^a^**	**65.4 ^a^**	**45.2 ^a^**	81.4	**74.8 ^a^**	**55.7 ^b^**	**70.0 ^b^**	69.5	94.3
	55 yrs +	**80.0 ^a^**	84.3	**77.1 ^a^**	75.2	**18.6 ^a^**	**66.7 ^a^**	**45.2 ^a^**	81.0	**52.9 ^b^**	**70.5 ^a^**	**80.0 ^a^**	69.5	95.7
	*p*-value	**<0.0001**	0.447	**0.007**	0.104	**0.003**	**0.005**	**0.017**	0.990	**<0.0001**	**0.005**	**<0.0001**	0.934	0.346
Sugar	18–34 yrs	**41.4 ^b^**	53.6	45.2	38.1	39.5	**44.5 ^b^**	69.0	**22.9 ^c^**	21.0	**20.0 ^c^**	**20.0 ^b^**	**16.7 ^b^**	20.0
	35–54 yrs	**43.8 ^b^**	47.6	42.9	40.0	49.5	**59.7 ^a^**	70.0	**44.3 ^b^**	21.0	**29.5 ^b^**	**35.2 ^a^**	**27.6 ^a^**	16.7
	55 yrs +	**65.7 ^a^**	51.4	47.1	47.6	50.0	**57.1 ^a^**	78.6	**56.7 ^a^**	20.5	**48.1 ^a^**	**42.4 ^a^**	**29.5 ^a^**	14.8
	*p*-value	**<0.0001**	0.468	0.677	0.112	0.052	**0.004**	0.055	**<0.0001**	0.990	**<0.0001**	**<0.0001**	**0.004**	0.354
Wheat flour	18–34 yrs	**59.5 ^b^**	84.8	60.5	71.0	63.3	63.6	**88.6 ^b^**	38.6	**72.9 ^a^**	**56.7 ^b^**	79.0	**66.7 ^b^**	76.2
	35–54 yrs	**53.8 ^b^**	87.6	59.0	70.5	69.5	62.1	**91.0 ^b^**	40.5	**81.4 ^a^**	**66.2 ^a^**	77.1	**70.5 ^b^**	72.4
	55 yrs +	**83.8 ^a^**	85.7	64.3	74.3	71.9	62.9	**96.2 ^a^**	48.6	**51.9 ^b^**	**71.9 ^a^**	71.4	**84.3 ^a^**	71.0
	*p*-value	**<0.0001**	0.701	0.523	0.639	0.150	0.947	**0.014**	0.089	**<0.0001**	**0.004**	0.165	**<0.0001**	0.457
Xanthan gum	18–34 yrs	8.6 ^ab^	4.3	1.0	6.7	**5.7 ^b^**	7.2	5.2	15.2	19.0	31.0	24.3	14.3	8.1
	35–54 yrs	6.2 ^b^	4.8	1.4	6.2	**12.4 ^a^**	8.5	5.7	16.7	15.7	31.4	17.1	12.9	12.9
	55 yrs +	11.9 ^a^	5.2	1.9	10.5	**12.9 ^a^**	9.0	3.3	17.1	11.4	31.0	22.4	11.9	8.1
	*p*-value	0.118	0.896	0.713	0.199	**0.027**	0.773	0.480	0.860	0.096	0.993	0.180	0.766	0.163

The data are presented in three columns: 18–34 years old, 35–54 years old, and more than 55 years old, and *p*-values are listed vertically. Ingredients with statistically significant differences (*p* < 0.05) according to the Kruskal–Wallis test are highlighted in bold. Different letters in percentages indicate statistically significant differences among the three age groups based on pairwise comparisons.

**Table 5:** Influence of education level on the perception of 20 ingredients as natural.

Ingredient	Education	Cluster 1						Cluster 2	Cluster 3					Cluster 4
		Australia	India	Japan	South Africa	United Kingdom	USA	Russia	Brazil	China	Mexico	Peru	Spain	Thailand
Baking soda	≤HS	19.5	26.7	0.9	**17.7**	22.9	35.0	1.8	18.9	14.2	10.7	8.2	15.9	**1.1**
	COL+	21.9	14.3	2.2	**10.0**	19.7	31.5	8.4	17.5	16.9	18.8	6.2	20.7	**6.3**
	*p*-value	0.473	0.064	0.233	**0.005**	0.349	0.354	0.080	0.654	0.445	0.283	0.534	0.123	**0.047**
BHA	≤HS	2.0	**16.7**	0.0	0.4	0.8	4.1	0.0	2.3	0.7	0.0	0.0	0.7	0.0
	COL+	2.4	**2.3**	0.2	1.3	2.1	3.6	0.2	1.3	1.2	1.5	0.7	0.6	1.3
	*p*-value	0.751	**<0.0001**	0.463	0.308	0.218	0.739	0.759	0.362	0.651	0.517	0.513	0.905	0.279
Black beans	≤HS	59.4	**36.7**	63.1	64.5	67.3	61.6	62.5	8.1	10.4	32.1	8.2	32.1	1.1
	COL+	62.8	**55.6**	62.3	70.2	64.7	63.7	65.5	8.1	12.7	23.8	10.5	39.5	1.1
	*p*-value	0.386	**0.043**	0.842	0.141	0.492	0.582	0.653	0.992	0.480	0.311	0.567	0.053	1.000
Canola oil	≤HS	44.2	33.3	28.4	63.6	**22.0**	40.1	10.7	45.9	47.8	**60.7**	**16.4**	9.5	14.4
	COL+	42.7	34.3	27.7	59.4	**31.2**	43.5	9.6	44.5	54.2	**39.7**	**31.6**	12.6	23.5
	*p*-value	0.714	0.916	0.856	0.294	**0.013**	0.401	0.785	0.715	0.183	**0.027**	**0.014**	0.215	0.055
Corn	≤HS	80.5	73.3	61.7	76.6	75.1	69.4	87.5	86.5	69.4	100.0	**80.3**	80.7	5.6
	COL+	78.9	83.4	61.5	77.2	70.4	68.8	84.7	81.1	76.2	91.9	**90.5**	79.6	2.8
	*p*-value	0.630	0.155	0.962	0.870	0.198	0.863	0.573	0.076	0.108	0.116	**0.014**	0.730	0.165
Corn syrup	≤HS	21.9	23.3	9.5	29.4	24.9	18.7	26.8	22.4	**20.9**	64.3	**44.3**	42.9	41.1
	COL+	22.2	21.8	9.1	24.6	28.8	20.2	27.0	22.6	**29.6**	45.5	**31.6**	46.1	49.6
	*p*-value	0.941	0.843	0.872	0.181	0.281	0.629	0.972	0.942	**0.045**	0.052	**0.046**	0.420	0.135
Gluten	≤HS	22.7	10.0	11.3	22.9	31.8	22.4	5.4	10.4	25.4	14.3	8.2	**18.9**	3.3
	COL+	28.5	12.8	14.7	20.3	31.7	23.5	10.5	12.4	29.0	14.5	16.9	**28.7**	8.0
	*p*-value	0.106	0.652	0.227	0.435	0.969	0.752	0.226	0.447	0.404	0.981	0.080	**0.004**	0.119
Insect powder	≤HS	5.2	10.0	13.5	3.9	**4.5**	**4.8**	19.6	10.8	10.4	28.6	6.6	9.1	3.3
	COL+	4.5	4.2	17.2	3.5	**8.8**	**9.2**	17.9	13.2	15.1	27.7	7.2	12.3	9.4
	*p*-value	0.690	0.130	0.232	0.803	**0.039**	**0.030**	0.753	0.367	0.169	0.924	0.853	0.203	0.055
Lecithin	≤HS	19.1	**16.7**	5.4	15.2	12.2	12.9	**5.4**	3.5	8.2	3.6	0.0	**2.4**	0.0
	COL+	23.0	**5.3**	6.1	16.3	13.5	14.3	**16.2**	5.7	7.3	3.8	0.9	**6.0**	1.1
	*p*-value	0.252	**0.010**	0.713	0.707	0.647	0.620	**0.031**	0.206	0.711	0.948	0.464	**0.025**	0.316
Maltodextrins	≤HS	5.2	3.3	1.4	**0.9**	4.9	4.1	5.4	37.8	**26.1**	25.0	70.5	31.1	60.0
	COL+	7.4	3.8	1.5	**4.3**	6.0	5.1	2.4	39.6	**46.6**	20.9	70.7	36.5	65.9
	*p*-value	0.272	0.892	0.905	**0.016**	0.566	0.560	0.199	0.652	**<0.0001**	0.607	0.980	0.150	0.275
Molasses	≤HS	38.2	16.7	**31.1**	41.6	39.6	46.6	5.4	1.5	7.5	0.0	0.0	1.4	0.0
	COL+	40.1	24.1	**40.0**	46.4	39.5	43.8	8.4	4.3	10.1	2.0	2.3	2.7	1.3
	*p*-value	0.641	0.350	**0.028**	0.243	0.978	0.474	0.432	0.051	0.360	0.452	0.234	0.237	0.279
Pea flour	≤HS	35.5	40.0	28.8	47.6	42.4	37.1	67.9	77.2	**55.2**	82.1	73.8	44.9	**81.1**
	COL+	40.4	50.1	30.6	51.6	41.6	39.0	69.3	77.6	**69.8**	86.9	81.0	49.4	**89.4**
	*p*-value	0.215	0.282	0.637	0.333	0.826	0.622	0.819	0.904	**0.002**	0.472	0.176	0.263	**0.023**
Salt	≤HS	54.6	76.7	48.6	60.2	56.3	55.4	80.4	33.2	26.9	71.4	47.5	59.8	**86.7**
	COL+	60.2	61.6	50.5	55.1	53.2	53.6	77.9	36.7	34.7	56.3	48.3	65.3	**94.8**
	*p*-value	0.166	0.096	0.659	0.219	0.450	0.639	0.669	0.373	0.088	0.115	0.907	0.157	**0.003**
SAPP	≤HS	2.4	**13.3**	1.4	1.7	2.4	3.7	1.8	2.7	0.7	0.0	0.0	0.3	2.2
	COL+	2.9	**3.5**	1.2	2.5	2.3	2.7	1.2	2.2	2.6	1.7	0.7	1.8	1.5
	*p*-value	0.699	**0.007**	0.894	0.526	0.930	0.449	0.720	0.659	0.192	0.494	0.513	0.082	0.604
Sodium bicarbonate	≤HS	16.7	**20.0**	3.6	9.5	15.5	12.6	39.3	**6.9**	**10.4**	35.7	11.5	12.2	4.4
	COL+	21.4	**5.3**	3.9	11.8	17.1	13.4	47.0	**12.9**	**5.0**	24.6	13.4	14.4	3.7
	*p*-value	0.151	**0.001**	0.843	0.383	0.591	0.764	0.267	**0.016**	**0.021**	0.185	0.680	0.416	0.735
Sorghum flour	≤HS	35.1	20.0	**0.5**	40.7	13.9	**19.4**	33.9	17.0	53.0	39.3	16.4	**15.5**	58.9
	COL+	37.2	27.1	**2.9**	48.1	15.8	**28.0**	31.9	19.1	62.3	35.2	23.0	**25.4**	64.1
	*p*-value	0.584	0.391	**0.036**	0.071	0.502	**0.012**	0.755	0.493	0.051	0.660	0.238	**0.002**	0.345
Soybean	≤HS	65.3	83.3	**66.7**	**64.9**	59.6	61.9	53.6	82.6	**56.0**	46.4	**50.8**	66.6	**88.9**
	COL+	71.2	81.7	**74.3**	**72.7**	58.4	61.3	40.1	80.1	**70.0**	62.6	**71.7**	73.1	**95.0**
	*p*-value	0.118	0.822	**0.044**	**0.041**	0.775	0.879	0.050	0.418	**0.002**	0.085	**0.001**	0.076	**0.023**
Sugar	≤HS	51.8	53.3	41.4	**47.2**	49.0	53.7	71.4	**35.1**	19.4	25.0	**16.4**	21.6	12.2
	COL+	49.3	50.7	47.1	**38.8**	44.7	53.9	72.6	**45.6**	21.2	32.9	**34.3**	27.2	18.0
	*p*-value	0.547	0.783	0.176	**0.041**	0.291	0.975	0.846	**0.009**	0.655	0.384	**0.005**	0.102	0.181
Wheat flour	≤HS	68.9	86.7	57.7	75.3	69.0	65.3	85.7	42.5	**56.7**	71.4	75.4	76.4	70.0
	COL+	63.6	86.0	63.2	69.9	67.8	60.7	92.5	42.6	**72.0**	64.6	75.9	71.6	73.7
	*p*-value	0.168	0.922	0.170	0.147	0.755	0.235	0.076	0.977	**0.001**	0.461	0.930	0.172	0.463
Xanthan gum	≤HS	**6.0**	6.7	0.5	5.2	7.8	8.8	5.4	15.8	**9.7**	35.7	**34.4**	10.8	7.8
	COL+	**10.8**	4.7	2.0	9.3	11.9	7.7	4.7	16.7	**16.9**	30.9	**19.9**	15.0	10.0
	*p*-value	**0.037**	0.615	0.128	0.066	0.092	0.616	0.827	0.769	**0.040**	0.591	**0.008**	0.122	0.510

The data are divided into two levels: ≤HS indicates primary school level or less or high school level or less, and COL+ indicates college or university level. Ingredients with statistically significant differences (*p* < 0.05) according to the Mann–Whitney U test are highlighted in bold.

**Table 6:** Influence of sex on the perception of 20 ingredients as natural.

Ingredient	Sex	Cluster 1						Cluster 2	Cluster 3					Cluster 4
		Australia	India	Japan	South Africa	United Kingdom	USA	Russia	Brazil	China	Mexico	Peru	Spain	Thailand
Baking soda	Male	20.0	14.3	1.3	13.0	22.6	31.8	**4.8**	16.5	17.1	16.2	5.4	**13.3**	4.4
	Female	21.9	15.5	2.2	12.7	19.3	34.5	**10.8**	19.7	15.6	20.6	7.3	**23.5**	6.7
	*p*-value	0.557	0.667	0.362	0.906	0.308	0.481	**0.005**	0.301	0.591	0.151	0.328	**0.001**	0.224
BHA	Male	2.9	3.2	0.3	1.0	1.3	5.1	0.0	1.3	**2.2**	1.6	**0.0**	0.3	1.3
	Female	1.6	2.8	0.0	1.0	1.9	2.5	0.3	2.2	**0.0**	1.3	**1.3**	1.0	1.0
	*p*-value	0.280	0.811	0.319	1.000	0.531	0.093	0.319	0.362	**0.008**	0.738	**0.045**	0.317	0.705
Black beans	Male	58.7	57.1	60.3	67.9	63.4	62.4	64.4	8.3	14.0	22.9	**6.7**	33.3	1.6
	Female	64.1	52.2	64.8	68.3	68.0	63.0	66.0	7.9	10.5	25.4	**14.0**	38.7	0.6
	*p*-value	0.165	0.214	0.250	0.932	0.218	0.886	0.676	0.884	0.181	0.457	**0.003**	0.159	0.255
Canola oil	Male	**48.9**	33.0	27.0	61.0	27.4	43.0	9.8	47.3	51.4	38.7	27.3	10.8	**18.7**
	Female	**37.8**	35.4	28.9	61.0	27.8	40.8	9.5	42.9	54.3	42.5	33.0	11.4	**25.7**
	*p*-value	**0.005**	0.521	0.595	1.000	0.898	0.581	0.893	0.263	0.473	0.331	0.119	0.800	**0.035**
Corn	Male	**76.2**	84.4	61.0	**80.3**	73.2	**73.9**	**81.9**	**87.6**	73.3	92.1	91.7	80.6	2.9
	Female	**82.9**	81.3	62.2	**73.7**	71.2	**64.2**	**87.9**	**79.0**	76.2	92.4	87.3	79.7	3.5
	*p*-value	**0.038**	0.299	0.744	**0.047**	0.567	**0.009**	**0.035**	**0.004**	0.410	0.882	0.069	0.765	0.650
Corn syrup	Male	23.5	23.5	9.8	27.9	29.6	**24.2**	26.0	**26.7**	25.4	49.5	35.6	45.4	49.8
	Female	20.6	20.3	8.6	24.8	25.0	**14.9**	27.9	**18.4**	30.2	43.2	30.2	43.8	47.0
	*p*-value	0.388	0.326	0.582	0.366	0.194	**0.003**	0.591	**0.013**	0.183	0.110	0.150	0.689	0.474
Gluten	Male	25.4	11.7	13.3	19.7	31.8	25.5	**7.0**	12.7	27.3	11.7	16.5	**20.0**	6.0
	Female	27.0	13.6	13.7	22.9	31.6	20.6	**13.0**	10.5	29.2	17.1	15.6	**28.3**	8.6
	*p*-value	0.651	0.483	0.908	0.331	0.957	0.144	**0.012**	0.384	0.596	0.054	0.745	**0.016**	0.221
Insect powder	Male	6.3	4.1	18.4	5.1	8.6	8.6	16.2	**14.9**	**10.5**	27.0	8.3	9.5	9.2
	Female	3.2	4.7	13.3	2.2	5.7	5.7	20.0	**9.5**	**17.8**	28.6	6.0	12.1	7.9
	*p*-value	0.062	0.706	0.081	0.056	0.158	0.158	0.215	**0.039**	**0.009**	0.657	0.279	0.305	0.570
Lecithin	Male	19.4	6.3	5.4	14.6	11.8	**16.9**	**9.8**	**2.5**	7.3	**2.2**	0.6	4.8	1.6
	Female	23.5	5.4	6.3	17.1	14.2	**10.4**	**20.6**	**7.0**	7.6	**5.4**	1.0	3.8	0.3
	*p*-value	0.207	0.605	0.612	0.384	0.360	**0.019**	**0.000**	**0.009**	0.880	**0.038**	0.655	0.556	0.101
Maltodextrins	Male	8.3	3.5	1.0	3.8	5.4	**6.7**	3.2	40.0	42.5	19.4	71.7	30.8	66.3
	Female	4.8	4.1	1.9	2.2	5.7	**2.5**	2.2	37.8	41.9	22.9	69.5	37.1	63.8
	*p*-value	0.076	0.684	0.315	0.245	0.878	**0.013**	0.462	0.568	0.872	0.283	0.541	0.093	0.504
Molasses	Male	38.1	25.1	**32.4**	45.1	37.9	46.2	8.6	2.9	8.3	2.5	1.3	**3.5**	0.6
	Female	40.6	22.5	**41.3**	44.1	41.1	44.0	7.6	3.5	10.8	1.3	2.9	**0.6**	1.6
	*p*-value	0.515	0.442	**0.021**	0.810	0.406	0.581	0.662	0.650	0.278	0.244	0.162	**0.012**	0.255
Pea flour	Male	39.0	49.8	31.1	48.3	40.4	40.8	67.0	**81.9**	66.3	88.3	80.0	48.9	87.9
	Female	37.8	49.4	28.9	52.1	43.4	35.4	71.4	**73.0**	67.0	85.1	80.6	45.7	88.6
	*p*-value	0.744	0.905	0.543	0.339	0.460	0.169	0.227	**0.008**	0.866	0.242	0.842	0.452	0.805
Salt	Male	61.0	**66.7**	51.1	**62.2**	57.3	56.1	79.7	**42.2**	32.1	59.7	47.9	64.8	92.1
	Female	54.9	**57.9**	48.6	**51.7**	51.6	52.8	76.5	**28.3**	34.0	54.3	48.6	60.6	95.2
	*p*-value	0.126	**0.023**	0.524	**0.008**	0.148	0.420	0.336	**0.000**	0.612	0.172	0.874	0.285	0.103
SAPP	Male	3.2	5.1	1.0	3.2	2.2	4.1	1.6	1.6	2.5	2.5	0.3	1.0	0.6
	Female	2.2	2.8	1.6	1.3	2.5	2.2	1.0	3.2	1.9	0.6	1.0	1.3	2.5
	*p*-value	0.462	0.151	0.478	0.105	0.804	0.169	0.478	0.192	0.590	0.056	0.317	0.705	0.056
Sodium bicarbonate	Male	18.4	5.1	3.5	12.1	16.2	15.6	43.8	9.8	6.7	**19.7**	**9.8**	**9.8**	4.4
	Female	20.6	7.0	4.1	9.8	16.8	10.4	48.9	11.1	5.7	**30.5**	**16.5**	**16.8**	3.2
	*p*-value	0.482	0.321	0.678	0.372	0.858	0.054	0.202	0.603	0.621	**0.002**	**0.013**	**0.010**	0.406
Sorghum flour	Male	39.0	28.3	1.9	**53.7**	**18.5**	**27.7**	30.5	**22.5**	61.0	**40.0**	**25.7**	18.1	65.4
	Female	33.7	25.3	2.2	**37.1**	**11.7**	**20.3**	33.7	**14.0**	59.7	**30.8**	**19.0**	23.5	61.3
	*p*-value	0.160	0.405	0.780	**<0.0001**	**0.018**	**0.029**	0.394	**0.005**	0.745	**0.016**	**0.045**	0.095	0.283
Soybean	Male	71.4	84.8	71.7	72.4	56.1	60.8	40.3	83.2	68.6	**57.5**	68.3	**65.1**	93.3
	Female	66.3	78.8	71.4	67.3	61.7	62.3	42.2	79.0	65.4	**66.3**	71.1	**74.9**	94.9
	*p*-value	0.169	0.053	0.930	0.165	0.149	0.697	0.628	0.186	0.397	**0.022**	0.436	**0.007**	0.398
Sugar	Male	53.7	**57.8**	48.6	**48.3**	**51.9**	57.3	75.9	40.3	19.7	31.7	34.6	**20.3**	16.8
	Female	47.0	**44.0**	41.6	**35.6**	**40.8**	50.3	69.2	42.2	21.9	33.3	30.5	**28.9**	17.5
	*p*-value	0.095	**0.001**	0.078	**0.001**	**0.005**	0.078	0.061	0.628	0.492	0.671	0.269	**0.013**	0.833
Wheat flour	Male	66.7	**88.9**	**65.4**	**78.1**	71.7	66.6	93.0	**52.4**	67.9	**72.1**	79.0	73.0	**76.8**
	Female	64.8	**83.2**	**57.1**	**65.7**	64.9	59.2	90.8	**32.7**	69.5	**57.8**	72.7	74.6	**69.5**
	*p*-value	0.615	**0.040**	**0.034**	**0.001**	0.068	0.055	0.307	**<0.0001**	0.668	**0.000**	0.063	0.651	**0.039**
Xanthan gum	Male	9.5	5.1	1.0	9.5	9.9	10.2	**2.5**	17.5	14.0	30.8	19.0	10.8	**12.1**
	Female	8.3	4.4	1.9	6.0	10.8	6.3	**7.0**	15.2	16.8	31.4	23.5	15.2	**7.3**
	*p*-value	0.576	0.702	0.315	0.102	0.715	0.079	**0.009**	0.451	0.321	0.864	0.173	0.098	**0.044**

Data are presented in three columns: male, female, and *p*-values listed vertically. Ingredients with statistically significant differences (*p* < 0.05) according to the Mann–Whitney U test are highlighted in bold.

**Table 7:** Influence of number of adults in the household on the perception of 20 ingredients as natural.

Ingredient	No. of Adults	Cluster 1						Cluster 2	Cluster 3					Cluster 4
		Australia	India	Japan	South Africa	United Kingdom	USA	Russia	Brazil	China	Mexico	Peru	Spain	Thailand
Baking soda	1–2	21.8	16.8	1.5	13.4	**22.9**	31.6	7.7	18.6	14.0	23.1	3.8	17.5	5.8
	3+	18.4	17.0	2.0	12.1	**14.8**	37.9	7.9	17.5	14.2	18.13	6.5	18.5	5.5
	*p*-value	0.379	0.933	0.613	0.618	**0.031**	0.141	0.954	0.733	0.951	0.439	0.594	0.838	0.884
BHA	1–2	2.5	**4.6**	0.0	0.8	1.9	3.8	0.0	1.8	0.7	2.6	**3.8**	0.0	1.0
	3+	1.3	**1.2**	0.3	1.1	0.6	3.7	0.4	1.7	2.4	1.4	**0.5**	0.7	1.1
	*p*-value	0.385	**0.008**	0.290	0.694	0.281	0.950	0.187	0.869	0.205	0.539	**0.036**	0.506	0.921
Black beans	1–2	62.8	**55.5**	61.7	**71.2**	67.6	64.0	63.1	8.8	**14.7**	30.8	3.8	36.5	1.6
	3+	57.2	**11.5**	63.5	**63.8**	60.0	59.0	69.0	7.3	**54.4**	23.7	0.5	36.0	0.9
	*p*-value	0.223	**<0.0001**	0.635	**0.048**	0.085	0.262	0.135	0.475	**<0.0001**	0.318	0.269	0.934	0.469
Canola oil	1–2	45.2	**34.7**	26.0	59.5	26.9	42.2	9.7	43.6	**51.0**	43.6	23.1	6.3	19.9
	3+	37.5	**53.4**	30.1	63.0	29.7	41.0	9.6	46.7	**34.1**	40.4	30.5	11.6	23.2
	*p*-value	0.096	**<0.0001**	0.262	0.366	0.510	0.786	0.962	0.436	**0.000**	0.699	0.422	0.205	0.355
Corn	1–2	79.9	80.9	59.6	76.2	74.1	69.9	85.5	84.5	**66.4**	92.3	84.6	73.0	2.1
	3+	78.3	77.2	63.9	78.1	66.5	66.5	83.8	82.1	**83.6**	92.2	89.7	81.0	3.6
	*p*-value	0.666	0.310	0.272	0.567	0.065	0.411	0.568	0.433	**<0.0001**	0.984	0.405	0.134	0.308
Corn syrup	1–2	23.6	**17.9**	7.8	28.2	28.4	20.3	25.7	**26.8**	23.8	53.8	30.8	47.6	50.3
	3+	17.1	**29.0**	10.8	23.8	23.9	17.4	29.3	**17.9**	23.4	45.9	32.9	44.3	47.6
	*p*-value	0.091	**0.005**	0.190	0.212	0.270	0.429	0.332	**0.007**	0.919	0.333	0.818	0.612	0.541
Gluten	1–2	26.8	**14.5**	12.9	**24.4**	32.0	24.1	10.0	12.2	**25.9**	23.1	11.5	27.0	4.7
	3+	24.3	**29.0**	14.2	**17.0**	31.0	19.9	10.0	10.9	**12.0**	13.9	16.2	23.8	8.4
	*p*-value	0.552	**0.000**	0.630	**0.025**	0.811	0.273	0.978	0.620	**<0.0001**	0.114	0.525	0.577	0.100
Insect powder	1–2	4.4	**5.2**	14.4	3.3	**8.8**	8.1	17.7	11.0	**11.2**	23.1	7.7	14.3	6.8
	3+	5.9	**15.0**	17.6	4.2	**1.9**	4.3	18.8	13.6	**4.1**	28.1	7.1	10.4	9.3
	*p*-value	0.442	**0.001**	0.274	0.569	**0.004**	0.111	0.737	0.320	**0.002**	0.499	0.913	0.347	0.297
Lecithin	1–2	22.6	6.9	7.2	**18.6**	**14.7**	14.3	15.2	4.3	5.6	2.6	0.0	3.2	2.1
	3+	17.8	8.0	4.4	**12.1**	**7.7**	11.8	15.3	5.3	5.5	3.9	0.8	4.4	0.5
	*p*-value	0.207	0.651	0.137	**0.026**	**0.025**	0.429	0.981	0.545	0.951	0.676	0.644	0.647	0.052
Maltodextrins	1–2	6.1	**3.5**	1.8	3.8	6.5	4.9	2.0	39.3	**30.8**	23.1	57.7	39.7	62.8
	3+	7.9	**45.6**	1.0	1.9	2.6	3.7	3.9	38.4	**3.9**	21.0	71.2	33.3	66.1
	*p*-value	0.427	**<0.0001**	0.410	0.159	0.063	0.539	0.150	0.814	**<0.0001**	0.757	0.139	0.313	0.435
Molasses	1–2	**41.8**	**24.3**	35.6	46.0	**43.6**	46.3	9.0	2.7	**8.4**	2.6	3.8	1.6	0.5
	3+	**31.6**	**9.9**	38.2	42.6	**27.1**	41.6	6.6	3.6	**23.6**	1.9	2.0	2.1	1.4
	*p*-value	**0.024**	**<0.0001**	0.509	0.399	**0.000**	0.306	0.283	0.521	**<0.0001**	0.757	0.516	0.781	0.354
Pea flour	1–2	38.9	**49.7**	31.4	52.3	43.8	**40.5**	67.6	78.7	55.2	84.6	69.2	49.2	86.9
	3+	36.8	**70.0**	28.4	47.2	36.1	**31.1**	72.1	76.2	49.6	86.8	80.8	47.1	88.8
	*p*-value	0.648	**<0.0001**	0.404	0.202	0.094	**0.033**	0.243	0.454	0.236	0.698	0.147	0.750	0.490
Salt	1–2	**60.5**	**65.9**	50.9	57.0	**57.9**	54.8	77.6	36.9	**25.9**	48.7	38.5	58.7	**90.1**
	3+	**50.0**	**35.1**	48.6	57.0	**43.9**	53.4	79.0	33.4	**60.9**	57.5	48.7	63.1	**95.2**
	*p*-value	**0.023**	**<0.0001**	0.573	0.999	**0.002**	0.762	0.666	0.366	**<0.0001**	0.282	0.308	0.493	**0.015**
SAPP	1–2	3.1	3.5	**2.1**	2.7	2.7	3.4	**0.5**	2.7	**0.7**	0.0	0.0	1.6	2.6
	3+	1.3	2.7	**0.3**	1.5	1.3	2.5	**2.6**	2.0	**4.1**	1.7	0.7	1.1	1.1
	*p*-value	0.228	0.590	**0.050**	0.302	0.306	0.564	**0.022**	0.534	**0.045**	0.414	0.680	0.706	0.173
Sodium bicarbonate	1–2	20.7	5.2	3.0	**13.4**	17.9	14.5	45.4	10.7	4.2	**38.5**	23.1	19.0	4.7
	3+	15.8	6.8	4.7	**7.5**	12.3	8.7	48.0	10.3	6.3	**24.2**	12.7	12.7	3.4
	*p*-value	0.183	0.467	0.257	**0.020**	0.101	0.059	0.522	0.868	0.342	**0.047**	0.128	0.160	0.436
Sorghum flour	1–2	**39.3**	**28.9**	2.4	45.8	16.0	25.4	32.9	17.4	**49.7**	33.3	11.5	17.5	**57.1**
	3+	**27.0**	**63.4**	1.7	44.9	12.3	19.9	30.6	19.2	**26.0**	35.5	22.8	21.2	**66.1**
	*p*-value	**0.006**	**<0.0001**	0.535	0.833	0.259	0.159	0.544	0.554	**<0.0001**	0.781	0.176	0.493	**0.032**
Soybean	1–2	70.5	**78.6**	70.4	69.3	**62.3**	62.5	42.4	81.1	**53.1**	69.2	76.9	65.1	92.7
	3+	63.8	71.0	73.0	70.6	**48.4**	59.0	39.3	81.1	**83.0**	61.4	69.4	70.5	94.8
	*p*-value	0.121	0.054	0.468	0.736	**0.002**	0.436	0.449	0.993	**<0.0001**	0.331	0.413	0.370	0.306
Sugar	1–2	52.3	**50.9**	44.6	41.4	**49.3**	54.6	71.6	43.0	**16.8**	35.9	26.9	23.8	18.8
	3+	44.1	**22.0**	45.6	42.6	**37.4**	51.6	74.2	39.4	**50.9**	32.3	32.8	24.7	16.4
	*p*-value	0.078	**<0.0001**	0.802	0.750	**0.010**	0.506	0.472	0.362	**<0.0001**	0.645	0.533	0.878	0.454
Wheat flour	1–2	67.8	**90.2**	59.9	70.1	68.6	63.5	91.0	43.9	**58.7**	74.4	69.2	77.8	70.7
	3+	59.2	**71.7**	62.8	74.3	67.1	60.9	93.4	41.1	**84.5**	64.3	76.2	73.4	74.3
	*p*-value	0.053	**<0.0001**	0.447	0.247	0.722	0.546	0.283	0.471	**<0.0001**	0.203	0.420	0.451	0.352
Xanthan gum	1–2	9.2	**3.5**	1.8	8.2	11.6	9.2	4.5	18.0	**11.2**	35.9	19.2	7.9	7.3
	3+	7.9	**16.6**	1.0	7.2	6.5	5.6	5.2	14.6	**5.2**	30.8	21.4	13.6	10.7
	*p*-value	0.622	**<0.0001**	0.410	0.628	0.069	0.155	0.671	0.247	**0.013**	0.506	0.796	0.207	0.188

Ingredients with statistically significant differences (*p* < 0.05) according to the Mann–Whitney U test are highlighted in bold.

**Table 8:** Influence of number of children in household on the perception of 20 ingredients as natural.

Ingredient	No. of Children	Cluster 1						Cluster 2	Cluster 3					Cluster 4
		Australia	India	Japan	South Africa	United Kingdom	USA	Russia	Brazil	China	Mexico	Peru	Spain	Thailand
Baking soda	None	21.7	16.5	1.5	13.9	20.7	34.9	6.1	20.3	15.3	18.9	7.7	18.2	6.6
	1+	19.4	13.8	2.5	11.7	21.4	29.6	9.5	15.3	17.1	18.1	5.5	18.7	4.6
	*p*-value	0.506	0.361	0.377	0.416	0.836	0.186	0.111	0.103	0.540	0.803	0.277	0.875	0.254
BHA	None	2.1	2.7	0.2	0.3	1.5	3.3	0.0	1.4	0.7	1.3	1.2	0.3	0.3
	1+	2.4	3.2	0.0	1.7	1.7	4.9	0.3	2.1	1.4	1.5	0.3	1.1	1.8
	*p*-value	0.859	0.748	0.567	0.067	0.810	0.340	0.302	0.504	0.453	0.783	0.144	0.175	0.075
Black beans	None	**68.3**	56.9	64.1	**73.2**	**70.3**	**66.3**	63.8	8.3	12.3	25.2	10.9	36.1	0.7
	1+	**47.9**	53.2	58.0	**62.2**	**57.6**	**55.3**	66.8	7.8	12.2	23.5	9.9	35.9	1.5
	*p*-value	**<0.0001**	0.364	0.172	**0.003**	**0.001**	**0.008**	0.434	0.827	0.952	0.621	0.705	0.946	0.307
Canola oil	None	**47.5**	33.7	28.3	59.9	27.2	42.2	10.1	44.1	49.6	37.4	27.0	10.3	22.9
	1+	**35.1**	34.6	26.8	62.2	28.4	41.3	9.2	46.3	55.2	42.6	32.2	12.2	21.6
	*p*-value	**0.003**	0.826	0.703	0.553	0.746	0.820	0.699	0.592	0.163	0.197	0.167	0.458	0.686
Corn	None	**84.0**	81.6	63.2	78.5	74.8	70.5	83.7	**80.2**	70.9	93.3	87.5	79.3	2.0
	1+	**70.6**	83.8	56.7	75.3	67.7	66.0	86.2	**87.2**	77.6	91.6	90.8	81.3	4.3
	*p*-value	**<0.0001**	0.470	0.146	0.341	0.055	0.252	0.393	**0.020**	0.055	0.442	0.182	0.546	0.106
Corn syrup	None	**26.0**	19.6	10.4	28.9	**30.2**	20.0	25.2	22.3	28.7	47.5	33.5	47.8	45.5
	1+	**14.2**	23.4	5.7	23.4	**22.3**	18.4	28.9	22.8	27.1	45.7	32.5	40.1	51.1
	*p*-value	**0.001**	0.258	0.083	0.116	**0.032**	0.635	0.284	0.899	0.646	0.658	0.793	0.054	0.164
Gluten	None	**31.3**	10.6	13.5	**25.7**	32.4	24.1	9.5	**14.6**	26.1	16.8	19.4	**27.2**	6.6
	1+	**16.1**	14.1	13.4	**16.2**	30.6	20.9	10.5	**7.8**	29.8	13.0	13.9	**19.8**	7.9
	*p*-value	**<0.0001**	0.194	0.961	**0.004**	0.632	0.374	0.671	**0.008**	0.306	0.189	0.067	**0.034**	0.545
Insect powder	None	**3.3**	3.1	16.1	2.4	7.0	7.3	19.6	12.9	11.9	31.5	7.7	10.3	7.0
	1+	**7.6**	5.3	15.3	5.2	7.4	6.8	16.4	11.4	15.7	25.5	6.8	11.5	10.0
	*p*-value	**0.018**	0.192	0.817	0.063	0.837	0.814	0.300	0.567	0.176	0.103	0.685	0.655	0.172
Lecithin	None	**27.7**	5.1	6.6	**20.6**	**15.5**	14.9	15.0	4.3	7.5	3.8	0.8	4.1	0.7
	1+	**9.0**	6.4	3.8	**10.3**	**8.7**	11.2	15.5	5.3	7.5	3.8	0.8	4.6	1.2
	*p*-value	**<0.0001**	0.501	0.208	**0.000**	**0.016**	0.206	0.881	0.543	0.999	0.978	0.978	0.759	0.478
Maltodextrins	None	6.9	2.7	1.7	2.7	6.5	4.7	2.5	38.4	**37.7**	20.2	70.2	35.3	65.4
	1+	5.7	4.5	0.6	3.4	3.9	4.4	3.0	39.5	**45.6**	21.7	70.9	32.1	64.7
	*p*-value	0.554	0.253	0.336	0.568	0.179	0.846	0.696	0.777	**0.048**	0.652	0.834	0.394	0.853
Molasses	None	**47.7**	**28.2**	36.6	**53.4**	**45.9**	**47.2**	8.9	3.2	11.2	1.7	2.0	1.6	**0.0**
	1+	**22.7**	**20.7**	37.6	**34.4**	**28.4**	40.8	7.2	3.2	8.3	2.0	2.1	2.7	**2.1**
	*p*-value	**<0.0001**	**0.030**	0.821	**<0.0001**	**<0.0001**	0.131	0.446	0.972	0.220	0.749	0.947	0.366	**0.011**
Pea flour	None	**41.8**	**54.9**	30.4	53.7	43.6	**40.8**	70.6	78.2	65.3	85.3	80.6	48.4	87.7
	1+	**31.8**	**46.0**	28.7	46.0	38.9	**32.5**	67.8	76.5	67.7	87.5	80.1	45.8	88.8
	*p*-value	**0.015**	**0.029**	0.673	0.056	0.243	**0.045**	0.449	0.610	0.531	0.430	0.868	0.525	0.684
Salt	None	**62.3**	66.3	50.3	60.2	**57.6**	54.5	79.1	37.8	32.8	58.4	52.4	63.9	92.7
	1+	**49.3**	59.6	48.4	53.3	**48.9**	54.4	77.0	32.0	33.1	56.1	45.5	61.1	94.5
	*p*-value	**0.002**	0.089	0.679	0.081	**0.035**	0.979	0.512	0.131	0.934	0.576	0.092	0.476	0.345
SAPP	None	2.4	3.1	1.1	1.5	2.0	3.3	**0.0**	2.0	1.1	1.7	1.2	0.8	1.0
	1+	3.3	4.5	1.9	3.1	3.1	2.9	**2.6**	2.8	3.0	1.5	0.3	1.5	2.1
	*p*-value	0.497	0.383	0.409	0.170	0.401	0.795	**0.003**	0.492	0.107	0.885	0.144	0.402	0.257
Sodium bicarbonate	None	**22.7**	5.5	4.0	12.4	**18.7**	14.4	45.7	11.5	4.1	25.6	16.1	14.9	3.3
	1+	**13.3**	6.4	3.2	9.3	**12.7**	10.2	47.0	9.3	7.7	24.7	11.3	11.1	4.3
	*p*-value	**0.005**	0.644	0.638	0.213	**0.050**	0.143	0.738	0.369	0.062	0.804	0.078	0.159	0.542
Sorghum flour	None	**43.7**	29.4	1.7	48.1	16.7	25.9	34.0	19.2	**55.6**	33.2	20.6	22.3	62.8
	1+	**21.8**	25.0	3.2	42.3	12.2	19.9	29.9	17.1	**63.8**	36.7	23.6	18.7	63.8
	*p*-value	**<0.0001**	0.220	0.255	0.144	0.131	0.096	0.269	0.495	**0.037**	0.368	0.379	0.276	0.787
Soybean	None	**74.5**	83.1	71.7	71.4	61.3	63.2	44.5	81.1	**60.8**	64.7	72.2	69.3	95.0
	1+	**57.8**	80.9	71.3	68.0	54.6	58.3	37.8	81.1	**71.5**	60.2	68.1	71.0	93.3
	*p*-value	**<0.0001**	0.466	0.936	0.362	0.097	0.231	0.091	0.988	**0.005**	0.260	0.273	0.647	0.364
Sugar	None	**58.0**	54.5	46.3	43.7	48.9	**57.8**	72.4	44.7	17.9	33.6	34.3	25.8	14.3
	1+	**35.1**	48.4	41.4	39.9	41.9	**45.6**	72.7	37.0	22.9	31.9	31.4	22.9	19.8
	*p*-value	**<0.0001**	0.133	0.286	0.336	0.092	**0.004**	0.932	0.052	0.125	0.654	0.455	0.403	0.069
Wheat flour	None	**72.6**	88.6	61.5	71.4	70.6	63.7	92.6	41.8	67.2	60.9	72.6	75.8	**68.8**
	1+	**52.1**	84.3	60.5	72.5	64.2	61.2	91.1	43.4	69.9	67.3	78.0	71.0	**77.2**
	*p*-value	**<0.0001**	0.125	0.822	0.755	0.098	0.541	0.485	0.690	0.466	0.102	0.120	0.175	**0.017**
Xanthan gum	None	9.8	5.1	1.5	**10.6**	**12.7**	8.0	3.7	14.6	13.8	29.8	19.4	12.5	8.0
	1+	7.1	4.5	1.3	**4.5**	**6.1**	8.7	5.9	18.5	16.6	31.9	22.5	13.7	11.2
	*p*-value	0.266	0.739	0.852	**0.004**	**0.009**	0.759	0.188	0.190	0.342	0.589	0.344	0.649	0.166

Ingredients with statistically significant differences (*p* < 0.05) according to the Mann–Whitney U test are highlighted in bold.

## Data Availability

The raw data supporting the conclusions of this study will be made available by the authors upon request.

## Abbreviations

The following abbreviations are used in this manuscript:

FDA	U.S. Food and Drug Admonition
WEIRD	Western, Educated, Industrialized, Rich, and Democratic
CATA	check-all-that-apply
TRAPD	translation, review, adjudication, pretesting, and documentation
BHA	butylated hydroxyanisole
UK	United Kingdom
USA	United States of America
SAPP	sodium acid pyrophosphate
GMO	genetically modified organism
GM	genetically modified
HFCS	high-fructose corn syrup
